# Synthesis and crystal structure of 2,4,6,8-tetra­kis­(3,5-di-*tert*-butyl­phen­oxy)pyrimido[5,4-*d*]pyrimidine: expansion of the Piedfort concept

**DOI:** 10.1107/S2056989019002470

**Published:** 2019-02-22

**Authors:** James H. Gall, David D. MacNicol, Ross MacSween, Christopher S. Frampton

**Affiliations:** a School of Chemistry, University of Glasgow, Glasgow, G12 8QQ, Scotland; b Experimental Techniques Centre, Brunel University London, Kingston Lane, Uxbridge, UB8 3PH, UK

**Keywords:** crystal structure, host–guest, inclusion, Piedfort, spider host, self-assembly

## Abstract

The title host compound, C_62_H_84_N_4_O_4_, designed to self-assemble to form a new type of extended core Piedfort unit reminiscent of an eight-legged spider host, forms a number of crystalline inclusion compounds favouring oxygen-containing guest mol­ecules. We have established the presence of this unit in the unsolvated mol­ecular crystal at 100 K, which is monoclinic, space group *P*2_1_/*n*, with *Z* = 8. The new Piedfort unit is chiral and its core structure closely approximates to *D*_2_ symmetry, with both enanti­omers present in the crystal. Rather than being superposed with a staggered arrangement of nitro­gen atoms, the rings are rotated by an angle of approximately 45° with respect to each other, and the shortest contact between them is 3.181 (2) Å. The compound’s significant inclusion properties may be taken to suggest the participation of an extended Piedfort unit in the microcrystalline adducts formed. The presence of such a dimeric host unit in the clathrates has, however, not yet been established because of the current lack of suitable single crystals for X-ray analysis.

## Abstract 2

The title host compound, designed to self-assemble to form a new type of extended core Piedfort unit reminiscent of an eight-legged spider host, forms a number of crystalline inclusion compounds favouring oxygen-containing guest mol­ecules. We have established the presence of this unit in the unsolvated mol­ecular crystal at 100 K, which is monoclinic, space group *P*2_1_/*n*, with *Z* = 8. The new Piedfort unit is chiral and its core structure closely approximates to *D*_2_ symmetry, with both enanti­omers present in the crystal. Rather than being superposed with a staggered arrangement of nitro­gen atoms, the rings are rotated by an angle of approximately 45° with respect to each other.

## Chemical context   

**Figure 1:**
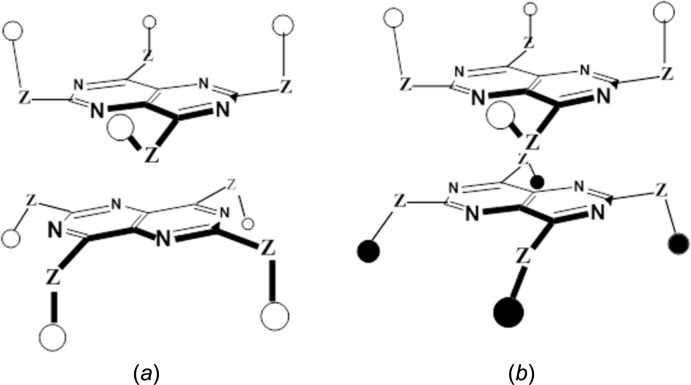
Alternative assembly modes for an extended Piedfort dimer, (uniform array of achiral side-chains where **Z** represents a link atom or chain. (*a*) one enanti­omer with idealized *D*_2_ symmetry. (*b*) idealized *C*_2_ *h* symmetry, side-chain groups residing in enanti­omerically related environments are distinguished by open and filled circle symbols.

Following its introduction in 1990 (Jessiman *et al.*, 1990 ▸) the Piedfort concept is now widely recognised to correspond to an effective supra­molecular synthon (Desiraju, 1995 ▸; Bombicz *et al.*, 2015 ▸ and references therein; Xu *et al.*, 2016 ▸; Mooibroek & Gamez, 2007 ▸; Saha *et al.*, 2005 ▸; Thalladi *et al.*, 1998 ▸). We employed this idea to construct a composite hexa­host mol­ecule (MacNicol, 1984 ▸). This was comprised of two juxtaposed mol­ecules of 2,4,6-tris­[4-(2-phenyl­propan-2-yl)phen­oxy]-1,3,5-triazine **1a**, and had exact *C*_i_ symmetry in both the unsolvated mol­ecular crystal and the 1,4-dioxane clathrate. Subsequently, Henderson *et al.* (1995 ▸) reported the *iso*-propanol clathrate of 2,4,6-tris­[4-(1-naphth­yl)phen­oxy]-1,3,5-triazine **1b**, also featuring a back-to-back arrangement of two tris­ubstituted 6π-electron aromatic rings. X-ray analysis revealed three types of Piedfort unit with respective symmetries *C*_3*i*_, *C*_3_ and *D*_3_, now designated as *C*_3i_-PU, *C*_3_-PU and *D*_3_-PU (Thalladi *et al.*, 1998 ▸). In the present work, considered even more challenging, we have sought to establish if a composite spider host (Downing & MacNicol, 1996 ▸) corresponding to an appropriately octa-substituted naphthalene could be produced using the extended 10 π-electron pyrimido[5,4-*d*]pyrimidine fused heterocyclic building block. The potential assembly of these building blocks is particularly inter­esting here since, unlike the 1,3,5-triazine core, the projected individual core component now has enanti­otopic faces. As illustrated in Fig. 1 ▸ *a*, idealized *D*_2_ is chiral, this symmetry being maintained for any angle of rotation about the vertical axis, whereas Fig. 1 ▸ *b*, *C*_2_ *h*, is achiral having a mirror plane (and inversion centre). This assembly mode with opposite enanti­otopic faces pointing outwards is also achiral by virtue of an improper axis of rotation, for a 90° component rotation (not shown) when the assembly has idealized *S*_4_ [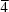] symmetry; for inter­mediate degrees of rotation between these extremes, however, enanti­omeric families with maximum *C*_2_ symmetry potentially exist. It is likely that the energy does not vary greatly (for link *Z* = O) among all these forms. This view is supported by the observation of situations significantly rotated away from a staggered arrangement in existing Piedfort units formed by 1,3,5-triazenes such as **1b** and **1c**, the latter unit, among others, has almost perfectly eclipsed nitro­gen atoms (Henderson *et al.*, 1995 ▸; Thalladi *et al.*, 1998 ▸). It was intriguing, therefore, to see what arrangement would be adopted by the new Piedfort unit if one could be produced.
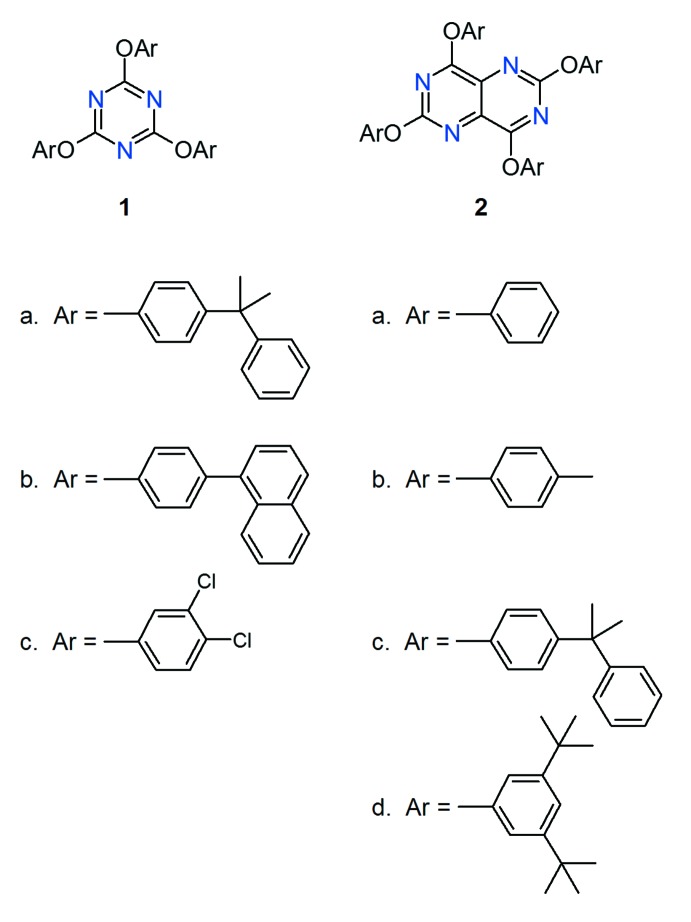


Candidate mol­ecules **2a**–**2d** were prepared, among many others which also had, in general, low solubility and high melting points (MacSween, 2004 ▸) by tetra-substitution of 2,4,6,8-tetra­chloro­pyrimido[5,4-*d*]pyrimidine (Fischer *et al.*, 1960 ▸), itself prepared from tetra­hydroxy­homopurine (Fischer & Roch, 1951 ▸), employing the appropriate sodium phenolate in THF. The structures as formulated were established employing ^1^H NMR, ^13^C NMR and MS data, as well as by single-crystal X-ray analysis for **2d**. It soon became clear, as indeed was anti­cipated, that a judicious choice of side chain would be critical. The parent mol­ecule **2a**, showed no host properties at all. The introduction of a single *meta*-methyl group to the side-chain rings of **2a**, to give **2b** (a tactic we have found effective in the spider series; Downing & MacNicol, 1996 ▸) also promoted no host properties and likewise **2c**, which shares a common side chain with host **1a**, showed no evidence of inclusion behaviour. Success was, however, achieved when two bulky *t*-butyl alkyl substituents were introduced onto the *meta* positions of the side-chain aromatic rings, as in **2d**. Compound **2d** proved to be a new host material forming crystalline inclusion compounds with, for example, DMF, acetone, THF, diethyl ether and diethyl carbonate, with common host–guest ratios of 2:1. We now report a single crystal analysis of the unsolvated crystal of **2d** which confirms the presence of the new, desired extended Piedfort unit. The formation of inclusion compounds by host **2d** is consistent with, and indeed may even be taken to suggest, the presence of the Piedfort unit in these microcrystalline adducts, however further work will be required to establish if this is in fact the case.

## Structural commentary   

**Figure 2:**
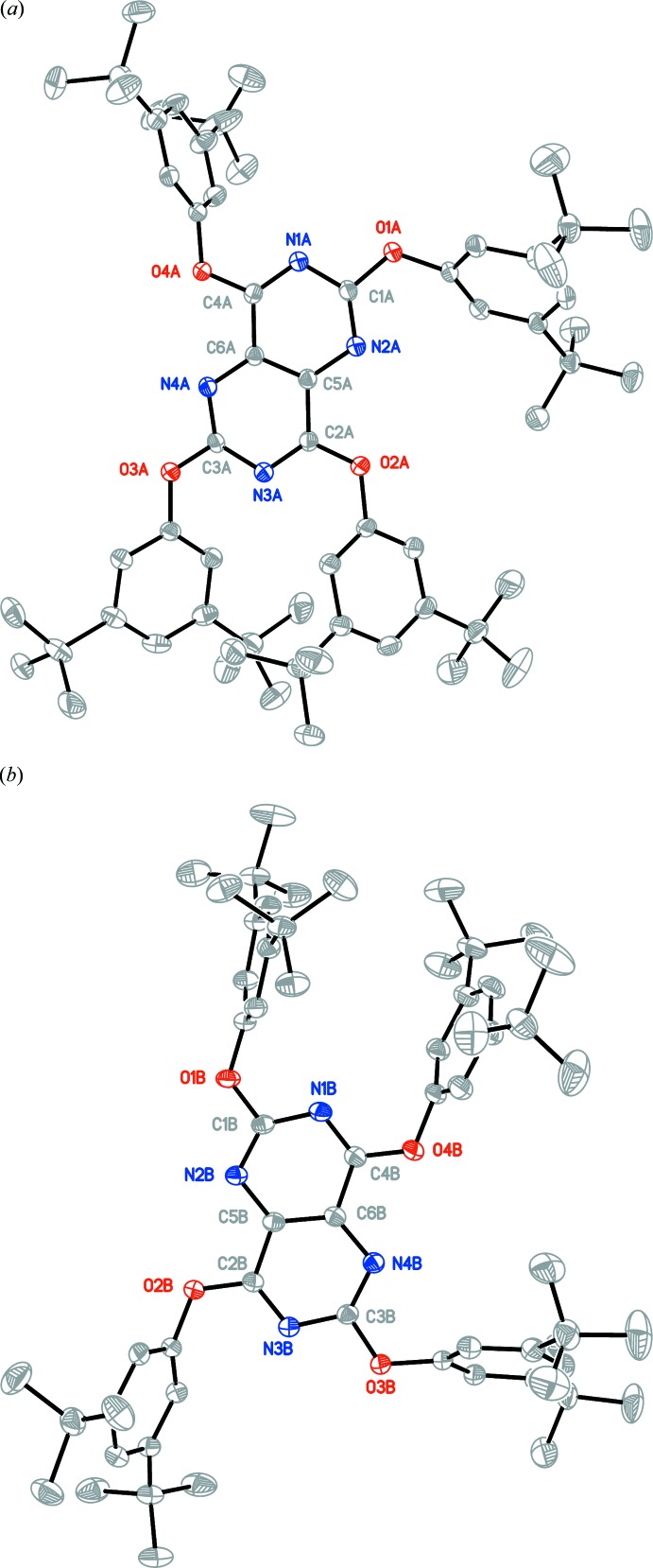
(*a*) View of mol­ecule *A* of the asymmetric unit with the atom labelling. Displacement ellipsoids are drawn at the 50% probability level. (*b*) View of mol­ecule *B* of the asymmetric unit with the atom labelling. Displacement ellipsoids are drawn at the 50% probability level.

**Figure 3:**
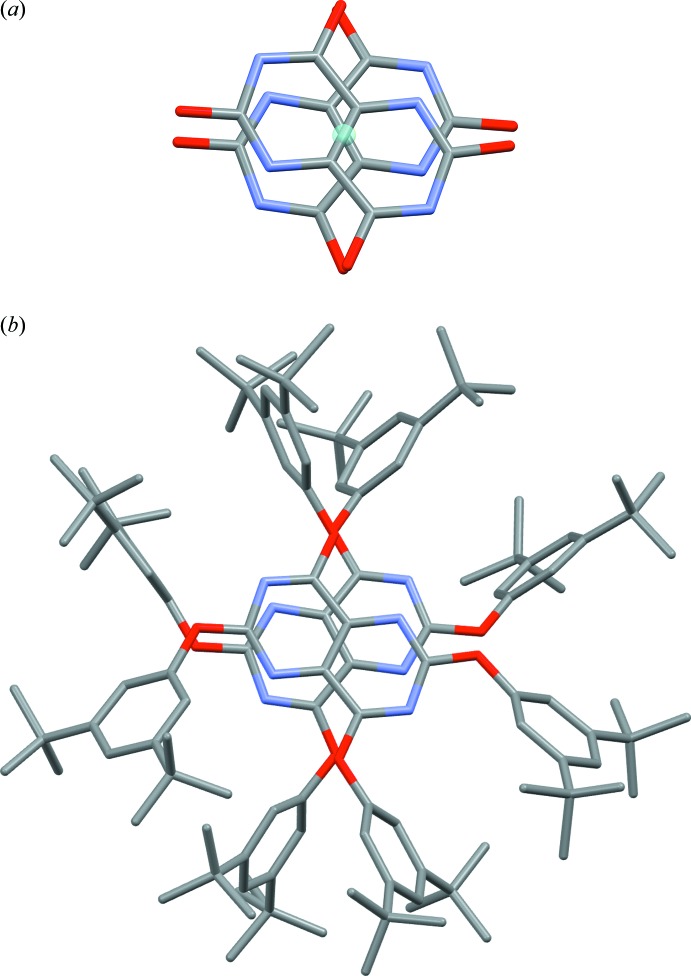
(*a*) View of the mol­ecule *A* and mol­ecule *B* pyrimido[5,4-*d*]pyrimidine cores, viewed down the overlapping C5*A*—C6*A* and C5*B*—C6*B* centroids; (*b*) as (*a*) with the side chains included.

Colourless block crystals of **2d** were obtained from CH_2_Br_2_/ethyl benzoate, (*ca* 1:5). The crystal structure is monoclinic, space group *P*2_1_/*n*, with two independent mol­ecules in the asymmetric unit (*Z*′ = 2). For clarity, each independent mol­ecule is labelled with the suffix *A* or *B*. It should be noted that six of the sixteen *t*-butyl alkyl substituents (three from mol­ecule *A* and three from mol­ecule *B*) exhibited rotational disorder, which was refined successfully with a two-part model. In cases where the disorder was severe, only an isotropic temperature factor was used for the disordered component. Fig. 2 ▸ *a* and 2*b* show displacement ellipsoid plots for the two mol­ecules, *A* and *B*. In these plots, the hydrogen atoms and the disordered components of the *t*-butyl alkyl substituents and atom labels for all atoms not present in the pyrimido[5,4-*d*]pyrimidine core have been omitted for clarity. The new extended Piedfort unit, comprised of the two mol­ecules in the asymmetric unit, is chiral and its core structure closely approximates to *D*_2_ symmetry, with both enanti­omers present in the crystal. This is exemplified by the values of the pseudo torsion angles N4*A*—C3*A*—N1*B*—C4*B*, −47.9 (1)°, N4*B*—C3*B*—N1*A*—C4*A*, −49.9 (1)°, N2*A*—C1*A*—N3*B*—C2*B*, −56.9 (1)°, N2*B*—C1*B*—N3*A*—C2*A*, −58.1 (1)°. The pyrimido[5,4-*d*]pyrimidine core units defined by the ten atoms N1, C1, N2, C5, C2, N3, C3, N4, C6 and C4 are approximately planar. A calculated least-squares plane through the ten atoms of the core gave r.m.s. deviations from planarity of 0.0333 and 0.0693 Å for mol­ecule *A* and mol­ecule *B*, respectively, and a calculated dihedral angle between them of 4.96 (3)°, showing them to be almost coplanar. The oxygen-atom displacements from the mean plane of the core of mol­ecule *A* are as follows: −0.127 (1), 0.017 (2), −0.125 (1) and 0.118 (2) Å for atoms O1*A* to O4*A* respectively. For the mean plane of the core of mol­ecule *B* the oxygen-atom displacements are −0.241 (1), 0.286 (2), −0.141 (2) and 0.339 (2) Å for atoms O1*B* to O4*B*, respectively. The core of mol­ecule *B* is markedly less planar than that of mol­ecule *A* and exhibits a twist about the central C—C bond C5*B*—C6*B* of 6.61 (6)°. When viewed down the overlapping centroids of the central C—C bonds, C5*A*—C6*A* and C5*B*—C6*B*, it can be seen that the two pyrimido[5,4-*d*]pyrimidine cores are rotated approximately 45° with respect to one another and that the shortest contact between the two cores is 3.181 (2) Å, see Fig. 3 ▸ *a* and 3*b*.

Finally, it is inter­esting to note that dimeric assembly of a suitable pyrimido[5,4-*d*]pyrimidine with four uniform homochiral side chains could produce two geometrically distinct (flexible) Piedfort *D*_2_ forms. Since these forms would not be enanti­omerically related, they would differ in stability and solubility, and preferential crystallization of just one of these two *D*_2_ forms might yield a novel chiral host lattice featuring potential amplification of chirality. Also, the successful production of benzene-based Piedfort units (Pigge *et al.*, 1999 ▸; Kumar *et al.*, 2004 ▸, Czugler *et al.*, 2003 ▸) suggests that carefully chosen 1,3,5,7-tetra­substituted naphthalenes might assemble to form composite spider hosts with enhanced solubility characteristics, although successful side-chain design would remain a formidable challenge.

## Supra­molecular features   

**Figure 4:**
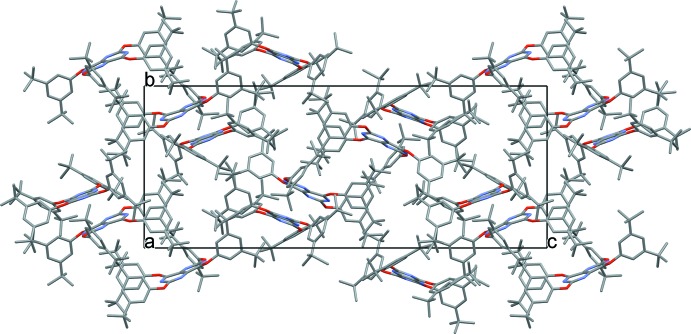
View of the crystal packing down the *a* axis.

A view of the crystal packing down the *a* axis is shown in Fig. 4 ▸. Given that there are no formal hydrogen-bond donors in the structure, the crystal packing between the dimers appears to be driven largely by van der Waals forces only. There are four notable C—H⋯O hydrogen bonds with H⋯O distances of less than 2.60 Å (Table 1 ▸).

## Database survey   

A search of the Cambridge Structural Database (CSD, Version 5.39 update August 2018; Groom *et al.*, 2016 ▸) for the pyrimido[5,4-*d*]pyrimidine core yielded just nine hits, all of which were genuine examples or analogues of the material under investigation. The nine hits divide into two distinct groups of mol­ecules. The first group is centred around the structural studies of the medication Dipyridamole, which is used to inhibit blood-clot formation. There are two structures of the freebase of Dipyridamole, BIRKES10 (Luger & Roch, 1983 ▸) and BIRKES01 (Codding & Jakana, 1984 ▸), which present data at 295 and 173 K, respectively. Structure QUQHER (Vepuri *et al.*, 2016 ▸) is a mono­hydro­chloride salt form of Dipyridamole solvated as a trihydrate. The final structure of this class, YUZBIE (López-Solera *et al.*, 1994 ▸), is a tris­(Dipyridamole) tetra­chloro­platinium(II) dihydrate analogue, which contains two protonated Diypridamole mol­ecules and a single mol­ecule of the freebase along with the tetra­chloro­platinium(II) counter-ion as a dihydrate solvate. The second group of mol­ecules is centred around structural studies of substituted 8-(β-d-ribo­furan­osyl­amino)­pyrimido[5,4-*d*]pyrimidines, which have been shown to exhibit novel anti-tumour behaviour. Structure KETTAE and its *s*-anomer KETSUX (Ghose *et al.*, 1990 ▸) are two examples of 4-meth­oxy-8-(β-d-ribo­furan­osyl­amino)­pyrimido[5,4-*d*]pyrimidine with both structures existing as monohydrate solvates. Structure RPPYPY20 (Narayanan & Berman, 1975 ▸) is a further example of a 4-substituted (4-amino) derivative. Structures KANZOO and KANZUU (Larson *et al.*, 1989 ▸) are examples of two substituted 8-2,3-*O*-iso­propyl­idene-β-d-ribo­furan­osyl­amino)­pyrimido-[5,4-*d*]pyrimidines, the substitutions being 2,4,6-tri­chloro and 4-amino-6-chloro, respectively. It is clear that the present structure is a unique example of the use of the pyrimido[5,4-*d*]pyrimidine core in the formation of a new class of potential host–guest compounds.

## Synthesis and crystallization   

Preparation of **2d**: Reaction of 2,4,6,8-tetra­chloro­pyrimido[5,4-*d*]pyrimidine (0.25 g, 0.93 mmol, 1eq), 3,5-di-*t*-butyl­phenol (1.24 g, 6.0 mmol, 6.5 eq) and sodium hydride (0.144 g, 6.0 mmol, 6.5 eq) in dry THF (15 mL) gave, following column chromatography on silicic acid (eluting with DCM), solvent removal under vacuum, and recrystallization from acetone gave the product as a solid gave the product as a crystalline solid, m.p. > 567 K, (0.25 g, 28.5%): ^1^H NMR (400 MHz, CDCl_3_) δ 7.14 (*t*, *J* = 1.6 Hz, 2H), 7.07 (*t*, *J* = 1.6 Hz, 2H), 7.01 (*d*, *J* = 1.6 Hz, 4H), 6.96 (*d*, *J* = 1.6 Hz, 4H), 1.16 (*s*, 36H), 1.15 (*s*, 36H). ^13^C NMR (100 MHz, CDCl_3_) δ 167.2, 159.9, 152.6, 152.3, 151.9, 151.8, 135.8, 120.0, 118.9, 115.8, 115.7, 35.3, 35.2, 31.7, 31.1; FAB MS, *m*/*z* 949.6 [*M* ^+^ + H], C_62_H_85_N_4_O_4_, calculated 949.7. Crystals suitable for X-ray diffraction studies were obtained by recrystallization from CH_2_Br_2_/ethyl benzoate (*ca* 1:5).

Compounds **2a**–**c** were prepared analogously in yields of 67, 33 and 72%, respectively; all had m.p. > 567 K, and ^1^H NMR, ^13^C NMR and MS data corresponding to their formulated structures. It is worthy of recording that the nature of the bulky substituents on the two *meta* positions is important since no evidence of host behaviour has yet been found for the (3,5-di-phen­yl)phen­oxy counterpart of **2d**, nor for 3,5-substitution by the smaller meth­oxy group (MacSween, 2004 ▸). Thus, at the present time the pyrimido­pyrimidine **2d** remains unique in displaying host properties.

## Refinement   

Crystal data, data collection and structure refinement details are summarized in Table 2 ▸. H atoms were placed in calculated positions and refined as riding with C—H = 0.95–0.98 Å and *U*_iso_(H) = 1.2–1.5*U*_eq_(C).

## Supplementary Material

Crystal structure: contains datablock(s) I. DOI: 10.1107/S2056989019002470/hb7803sup1.cif

Structure factors: contains datablock(s) I. DOI: 10.1107/S2056989019002470/hb7803Isup2.hkl

Supporting information file. DOI: 10.1107/S2056989019002470/hb7803Isup3.cml

CCDC reference: 1897649

Additional supporting information:  crystallographic information; 3D view; checkCIF report

## Figures and Tables

**Table 1:** Hydrogen-bond geometry (Å, °)

*D*—H⋯*A*	*D*—H	H⋯*A*	*D*⋯*A*	*D*—H⋯*A*
C48*A*—H48*A*⋯O1*B*	0.95	2.47	3.3187 (17)	149
C50*A*—H50*A*⋯O4*B*	0.95	2.56	3.4413 (18)	154
C34*B*—H34*B*⋯O2*A*	0.95	2.55	3.3259 (18)	139
C36*B*—H36*B*⋯O1*A*	0.95	2.48	3.4101 (18)	165

**Table 2:** Experimental details

Crystal data	
Chemical formula	C_62_H_84_N_4_O_4_
*M*_r_	949.33
Crystal system, space group	Monoclinic, *P*2_1_/*n*
Temperature (K)	100
*a*, *b*, *c* (Å)	18.48641 (17), 15.84310 (14), 39.4611 (4)
β (°)	93.0666 (8)
*V* (Å^3^)	11540.91 (18)
*Z*	8
Radiation type	Cu *K*α
μ (mm^−1^)	0.52
Crystal size (mm)	0.22 × 0.15 × 0.07

Data collection	
Diffractometer	Rigaku Oxford Diffraction SuperNova, Dualflex, AtlasS2
Absorption correction	Gaussian (*CrysAlis PRO*; Rigaku OD, 2015 ▸)
*T*_min_, *T*_max_	0.931, 0.971
No. of measured, independent and observed [*I* > 2σ(*I*)] reflections	55767, 23570, 19544
*R*_int_	0.024
(sin θ/λ)_max_ (Å^−1^)	0.625

Refinement	
*R*[*F* ^2^ > 2σ(*F* ^2^)], *wR*(*F* ^2^), *S*	0.048, 0.130, 1.04
No. of reflections	23570
No. of parameters	1461
H-atom treatment	H-atom parameters constrained
Δρ_max_, Δρ_min_ (e Å^−3^)	0.41, −0.32

Computer programs: *CrysAlis PRO* (Rigaku OD, 2015 ▸), *SHELXD2014* (Schneider & Sheldrick, 2002 ▸), *SHELXL2014* (Sheldrick, 2015 ▸), *SHELXTL* (Sheldrick, 2015 ▸), *SHELXTL* (Sheldrick, 2008 ▸), *Mercury* (Macrae *et al.*, 2008 ▸) and *publCIF* (Westrip, 2010 ▸).

## supplementary crystallographic information

### Crystal data

**Table:**

C_62_H_84_N_4_O_4_	*F*(000) = 4128
*M**_r_* = 949.33	*D*_x_ = 1.093 Mg m^−^^3^
Monoclinic, *P*2_1_/*n*	Cu *K*α radiation, λ = 1.54184 Å
*a* = 18.48641 (17) Å	Cell parameters from 22911 reflections
*b* = 15.84310 (14) Å	θ = 2.2–76.3°
*c* = 39.4611 (4) Å	µ = 0.52 mm^−^^1^
β = 93.0666 (8)°	*T* = 100 K
*V* = 11540.91 (18) Å^3^	Block, colourless
*Z* = 8	0.22 × 0.15 × 0.07 mm

### Data collection

**Table:**

Rigaku Oxford Diffraction SuperNova, Dualflex, AtlasS2 diffractometer	23570 independent reflections
Radiation source: fine-focus sealed X-ray tube, Enhance (Cu) X-ray Source	19544 reflections with *I* > 2σ(*I*)
Detector resolution: 5.2921 pixels mm^-1^	*R*_int_ = 0.024
ω scans	θ_max_ = 74.5°, θ_min_ = 2.2°
Absorption correction: gaussian (CrysAlis PRO; Rigaku OD, 2015)	*h* = −22→23
*T*_min_ = 0.931, *T*_max_ = 0.971	*k* = −19→18
55767 measured reflections	*l* = −49→35

### Refinement

**Table:**

Refinement on *F*^2^	0 restraints
Least-squares matrix: full	Hydrogen site location: inferred from neighbouring sites
*R*[*F*^2^ > 2σ(*F*^2^)] = 0.048	H-atom parameters constrained
*wR*(*F*^2^) = 0.130	*w* = 1/[σ^2^(*F*_o_^2^) + (0.0575*P*)^2^ + 4.150*P*] where *P* = (*F*_o_^2^ + 2*F*_c_^2^)/3
*S* = 1.04	(Δ/σ)_max_ = 0.001
23570 reflections	Δρ_max_ = 0.41 e Å^−^^3^
1461 parameters	Δρ_min_ = −0.32 e Å^−^^3^

### Special details

**Table:**

Geometry. All esds (except the esd in the dihedral angle between two l.s. planes) are estimated using the full covariance matrix. The cell esds are taken into account individually in the estimation of esds in distances, angles and torsion angles; correlations between esds in cell parameters are only used when they are defined by crystal symmetry. An approximate (isotropic) treatment of cell esds is used for estimating esds involving l.s. planes.

### Fractional atomic coordinates and isotropic or equivalent isotropic displacement parameters (Å^2^)

**Table:**

	*x*	*y*	*z*	*U*_iso_*/*U*_eq_	Occ. (<1)
O1A	0.18267 (5)	0.23374 (6)	0.30046 (2)	0.0245 (2)	
O2A	0.44643 (5)	0.22229 (6)	0.28634 (2)	0.02371 (19)	
O3A	0.52719 (5)	0.11187 (7)	0.39035 (2)	0.0251 (2)	
O4A	0.27392 (5)	0.14668 (7)	0.40357 (2)	0.0248 (2)	
N1A	0.22709 (6)	0.18928 (7)	0.35087 (3)	0.0216 (2)	
N2A	0.30715 (6)	0.22284 (7)	0.30665 (3)	0.0220 (2)	
N3A	0.49080 (6)	0.17001 (7)	0.33815 (3)	0.0211 (2)	
N4A	0.41041 (6)	0.14137 (7)	0.38251 (3)	0.0202 (2)	
C1A	0.24405 (7)	0.21479 (8)	0.31926 (3)	0.0212 (2)	
C2A	0.43673 (7)	0.19706 (8)	0.31823 (3)	0.0205 (2)	
C3A	0.47390 (7)	0.14269 (8)	0.36926 (3)	0.0196 (2)	
C4A	0.28195 (7)	0.16923 (8)	0.37138 (3)	0.0205 (2)	
C5A	0.36357 (7)	0.19836 (8)	0.32830 (3)	0.0199 (2)	
C6A	0.35559 (7)	0.16968 (8)	0.36120 (3)	0.0196 (2)	
C7A	0.18650 (7)	0.26279 (9)	0.26690 (3)	0.0227 (3)	
C8A	0.15401 (8)	0.33989 (9)	0.26016 (3)	0.0251 (3)	
H8AA	0.1364	0.3728	0.2781	0.030*	
C9A	0.14730 (8)	0.36904 (9)	0.22687 (3)	0.0252 (3)	
C10A	0.11194 (9)	0.45485 (10)	0.21917 (4)	0.0322 (3)	
C11A	0.04529 (14)	0.46700 (16)	0.23980 (7)	0.0713 (8)	
H11A	0.0214	0.5202	0.2332	0.107*	
H11B	0.0600	0.4687	0.2640	0.107*	
H11C	0.0116	0.4200	0.2354	0.107*	
C12A	0.08768 (13)	0.46340 (11)	0.18175 (5)	0.0494 (5)	
H12A	0.1303	0.4640	0.1680	0.074*	
H12B	0.0606	0.5161	0.1783	0.074*	
H12C	0.0566	0.4156	0.1750	0.074*	
C13A	0.16719 (13)	0.52314 (11)	0.22866 (6)	0.0557 (6)	
H13A	0.2073	0.5200	0.2135	0.084*	
H13B	0.1858	0.5147	0.2521	0.084*	
H13C	0.1441	0.5787	0.2265	0.084*	
C14A	0.17618 (8)	0.31998 (9)	0.20160 (3)	0.0249 (3)	
H14A	0.1726	0.3399	0.1789	0.030*	
C15A	0.21016 (7)	0.24273 (9)	0.20838 (3)	0.0232 (3)	
C16A	0.23903 (8)	0.18808 (9)	0.18015 (3)	0.0271 (3)	
C17A	0.19191 (10)	0.10924 (11)	0.17642 (5)	0.0408 (4)	
H17A	0.1958	0.0767	0.1976	0.061*	
H17B	0.2083	0.0746	0.1578	0.061*	
H17C	0.1413	0.1258	0.1716	0.061*	
C18A	0.23649 (10)	0.23435 (11)	0.14586 (4)	0.0398 (4)	
H18A	0.1861	0.2470	0.1387	0.060*	
H18B	0.2578	0.1985	0.1288	0.060*	
H18C	0.2640	0.2871	0.1482	0.060*	
C19A	0.31823 (9)	0.16354 (11)	0.18866 (4)	0.0368 (4)	
H19A	0.3220	0.1351	0.2107	0.055*	
H19B	0.3484	0.2144	0.1895	0.055*	
H19C	0.3348	0.1254	0.1711	0.055*	
C20A	0.21427 (7)	0.21347 (9)	0.24186 (3)	0.0230 (3)	
H20A	0.2359	0.1604	0.2473	0.028*	
C21A	0.51637 (7)	0.22621 (9)	0.27375 (3)	0.0243 (3)	
C22A	0.52289 (8)	0.19026 (9)	0.24210 (3)	0.0266 (3)	
H22A	0.4831	0.1615	0.2311	0.032*	
C23A	0.58873 (8)	0.19696 (10)	0.22657 (4)	0.0294 (3)	
C24A	0.59939 (9)	0.15834 (11)	0.19141 (4)	0.0365 (4)	
C25A	0.65690 (12)	0.08860 (15)	0.19478 (5)	0.0566 (5)	
H25A	0.7026	0.1125	0.2042	0.085*	
H25B	0.6643	0.0643	0.1724	0.085*	
H25C	0.6406	0.0444	0.2100	0.085*	
C26A	0.53010 (12)	0.11773 (17)	0.17597 (5)	0.0597 (6)	
H26A	0.4919	0.1605	0.1735	0.089*	
H26B	0.5146	0.0725	0.1909	0.089*	
H26C	0.5395	0.0942	0.1537	0.089*	
C27A	0.62394 (16)	0.22626 (15)	0.16737 (5)	0.0660 (7)	
H27A	0.5871	0.2707	0.1652	0.099*	
H27B	0.6308	0.2014	0.1450	0.099*	
H27C	0.6698	0.2505	0.1764	0.099*	
C28A	0.64560 (8)	0.23976 (10)	0.24404 (4)	0.0307 (3)	
H28A	0.6909	0.2437	0.2339	0.037*	
C29A	0.63838 (8)	0.27678 (10)	0.27573 (4)	0.0280 (3)	
C30A	0.69893 (8)	0.32765 (11)	0.29442 (4)	0.0336 (3)	
C31A	0.77248 (9)	0.31503 (14)	0.27906 (5)	0.0470 (4)	
H31A	0.7691	0.3320	0.2551	0.070*	
H31B	0.7864	0.2554	0.2808	0.070*	
H31C	0.8090	0.3496	0.2914	0.070*	
C32A	0.67911 (10)	0.42176 (12)	0.29197 (6)	0.0491 (5)	
H32A	0.6742	0.4385	0.2681	0.074*	
H32B	0.7173	0.4553	0.3037	0.074*	
H32C	0.6331	0.4314	0.3026	0.074*	
C33A	0.70619 (10)	0.30204 (13)	0.33206 (4)	0.0433 (4)	
H33A	0.7154	0.2412	0.3338	0.065*	
H33B	0.6612	0.3156	0.3430	0.065*	
H33C	0.7465	0.3330	0.3434	0.065*	
C34A	0.57167 (8)	0.26991 (9)	0.29073 (3)	0.0256 (3)	
H34A	0.5647	0.2949	0.3122	0.031*	
C35A	0.59885 (7)	0.10245 (9)	0.38112 (3)	0.0231 (3)	
C36A	0.61709 (8)	0.05950 (9)	0.35223 (4)	0.0266 (3)	
H36A	0.5805	0.0399	0.3363	0.032*	
C37A	0.68989 (9)	0.04541 (10)	0.34681 (4)	0.0317 (3)	
C38A	0.71261 (10)	−0.00036 (12)	0.31477 (5)	0.0427 (4)	0.589 (3)
C39A	0.6466 (2)	−0.0187 (2)	0.28881 (8)	0.0476 (9)	0.589 (3)
H39A	0.6132	−0.0582	0.2990	0.071*	0.589 (3)
H39B	0.6644	−0.0435	0.2681	0.071*	0.589 (3)
H39C	0.6212	0.0342	0.2832	0.071*	0.589 (3)
C40A	0.7599 (2)	0.0667 (2)	0.29347 (8)	0.0486 (9)	0.589 (3)
H40A	0.8038	0.0823	0.3070	0.073*	0.589 (3)
H40B	0.7309	0.1173	0.2884	0.073*	0.589 (3)
H40C	0.7734	0.0406	0.2722	0.073*	0.589 (3)
C41A	0.7547 (2)	−0.0747 (2)	0.32205 (8)	0.0479 (9)	0.589 (3)
H41A	0.7256	−0.1155	0.3341	0.072*	0.589 (3)
H41B	0.7978	−0.0598	0.3363	0.072*	0.589 (3)
H41C	0.7694	−0.0997	0.3008	0.072*	0.589 (3)
C38C	0.71261 (10)	−0.00036 (12)	0.31477 (5)	0.0427 (4)	0.411 (3)
C39C	0.6730 (3)	−0.0944 (3)	0.31624 (10)	0.0416 (11)	0.411 (3)
H39D	0.6203	−0.0871	0.3154	0.062*	0.411 (3)
H39E	0.6888	−0.1230	0.3374	0.062*	0.411 (3)
H39F	0.6865	−0.1283	0.2968	0.062*	0.411 (3)
C40C	0.6930 (3)	0.0419 (3)	0.28613 (10)	0.0407 (11)	0.411 (3)
H40D	0.7183	0.0963	0.2861	0.061*	0.411 (3)
H40E	0.6406	0.0516	0.2851	0.061*	0.411 (3)
H40F	0.7060	0.0087	0.2664	0.061*	0.411 (3)
C41C	0.7953 (3)	−0.0282 (4)	0.31851 (12)	0.0492 (13)	0.411 (3)
H41D	0.8262	0.0221	0.3192	0.074*	0.411 (3)
H41E	0.8068	−0.0634	0.2991	0.074*	0.411 (3)
H41F	0.8039	−0.0604	0.3396	0.074*	0.411 (3)
C42A	0.74183 (8)	0.07407 (10)	0.37120 (4)	0.0338 (3)	
H42A	0.7916	0.0641	0.3677	0.041*	
C43A	0.72341 (8)	0.11673 (9)	0.40041 (4)	0.0295 (3)	
C44A	0.78140 (9)	0.14691 (11)	0.42696 (5)	0.0370 (4)	0.487 (3)
C45A	0.8346 (2)	0.2013 (3)	0.41118 (10)	0.0548 (13)	0.487 (3)
H45A	0.8097	0.2499	0.4005	0.082*	0.487 (3)
H45B	0.8592	0.1692	0.3940	0.082*	0.487 (3)
H45C	0.8704	0.2213	0.4286	0.082*	0.487 (3)
C46A	0.7497 (2)	0.1965 (2)	0.45767 (9)	0.0426 (9)	0.487 (3)
H46A	0.7241	0.2469	0.4491	0.064*	0.487 (3)
H46B	0.7895	0.2132	0.4737	0.064*	0.487 (3)
H46C	0.7160	0.1599	0.4692	0.064*	0.487 (3)
C47A	0.82148 (19)	0.0679 (3)	0.44520 (9)	0.0398 (9)	0.487 (3)
H47A	0.8436	0.0328	0.4281	0.060*	0.487 (3)
H47B	0.7861	0.0343	0.4570	0.060*	0.487 (3)
H47C	0.8592	0.0883	0.4616	0.060*	0.487 (3)
C44C	0.78140 (9)	0.14691 (11)	0.42696 (5)	0.0370 (4)	0.513 (3)
C45C	0.85762 (19)	0.1263 (3)	0.41457 (13)	0.0594 (13)	0.513 (3)
H45D	0.8626	0.0651	0.4119	0.089*	0.513 (3)
H45E	0.8947	0.1468	0.4313	0.089*	0.513 (3)
H45F	0.8638	0.1541	0.3927	0.089*	0.513 (3)
C46C	0.77503 (18)	0.2455 (2)	0.42846 (9)	0.0396 (8)	0.513 (3)
H46D	0.7280	0.2611	0.4369	0.059*	0.513 (3)
H46E	0.7792	0.2690	0.4057	0.059*	0.513 (3)
H46F	0.8139	0.2681	0.4437	0.059*	0.513 (3)
C47C	0.7679 (2)	0.1083 (3)	0.45883 (10)	0.0543 (11)	0.513 (3)
H47D	0.7697	0.0468	0.4565	0.081*	0.513 (3)
H47E	0.7199	0.1252	0.4658	0.081*	0.513 (3)
H47F	0.8049	0.1265	0.4760	0.081*	0.513 (3)
C48A	0.65031 (8)	0.13140 (9)	0.40501 (4)	0.0253 (3)	
H48A	0.6359	0.1611	0.4244	0.030*	
C49A	0.20381 (7)	0.13319 (9)	0.41511 (3)	0.0241 (3)	
C50A	0.17711 (8)	0.19182 (9)	0.43682 (3)	0.0256 (3)	
H50A	0.2020	0.2435	0.4412	0.031*	
C51A	0.11258 (8)	0.17412 (10)	0.45242 (3)	0.0277 (3)	
C52A	0.08398 (8)	0.23447 (11)	0.47896 (4)	0.0315 (3)	
C53A	0.00131 (10)	0.23397 (16)	0.47822 (5)	0.0555 (6)	
H53A	−0.0158	0.1768	0.4828	0.083*	
H53B	−0.0181	0.2522	0.4558	0.083*	
H53C	−0.0153	0.2726	0.4956	0.083*	
C54A	0.10897 (12)	0.32502 (12)	0.47340 (5)	0.0498 (5)	
H54A	0.1618	0.3280	0.4768	0.075*	
H54B	0.0866	0.3625	0.4896	0.075*	
H54C	0.0945	0.3427	0.4502	0.075*	
C55A	0.11374 (12)	0.20532 (15)	0.51400 (4)	0.0509 (5)	
H55A	0.1668	0.2072	0.5149	0.076*	
H55B	0.0978	0.1474	0.5181	0.076*	
H55C	0.0957	0.2427	0.5315	0.076*	
C56A	0.07857 (9)	0.09755 (11)	0.44476 (4)	0.0353 (3)	
H56A	0.0347	0.0848	0.4552	0.042*	
C57A	0.10580 (9)	0.03845 (11)	0.42243 (4)	0.0374 (4)	
C58A	0.06584 (14)	−0.04477 (15)	0.41535 (7)	0.0739 (9)	0.440 (11)
C59A	0.0763 (5)	−0.0985 (3)	0.45552 (16)	0.0496 (18)	0.440 (11)
H59A	0.0527	−0.0657	0.4729	0.074*	0.440 (11)
H59B	0.1279	−0.1046	0.4620	0.074*	0.440 (11)
H59C	0.0538	−0.1544	0.4536	0.074*	0.440 (11)
C60A	−0.0028 (3)	−0.0437 (4)	0.4067 (3)	0.058 (2)	0.440 (11)
H60A	−0.0104	−0.0166	0.3844	0.087*	0.440 (11)
H60B	−0.0286	−0.0119	0.4236	0.087*	0.440 (11)
H60C	−0.0212	−0.1016	0.4055	0.087*	0.440 (11)
C61A	0.10872 (13)	−0.10583 (12)	0.39423 (5)	0.0532 (5)	0.440 (11)
H61A	0.1141	−0.0819	0.3716	0.080*	0.440 (11)
H61B	0.0829	−0.1598	0.3921	0.080*	0.440 (11)
H61C	0.1567	−0.1150	0.4054	0.080*	0.440 (11)
C58C	0.06584 (14)	−0.04477 (15)	0.41535 (7)	0.0739 (9)	0.560 (11)
C59C	0.0331 (5)	−0.0794 (4)	0.44315 (17)	0.073 (2)	0.560 (11)
H59D	0.0703	−0.0928	0.4610	0.109*	0.560 (11)
H59E	0.0073	−0.1311	0.4362	0.109*	0.560 (11)
H59F	−0.0013	−0.0387	0.4518	0.109*	0.560 (11)
C60C	−0.0078 (2)	−0.0109 (5)	0.38788 (18)	0.0602 (17)	0.560 (11)
H60D	−0.0353	0.0326	0.3992	0.090*	0.560 (11)
H60E	−0.0395	−0.0591	0.3822	0.090*	0.560 (11)
H60F	0.0108	0.0122	0.3670	0.090*	0.560 (11)
C61C	0.10872 (13)	−0.10583 (12)	0.39423 (5)	0.0532 (5)	0.560 (11)
H61D	0.1539	−0.1213	0.4069	0.080*	0.560 (11)
H61E	0.1200	−0.0787	0.3728	0.080*	0.560 (11)
H61F	0.0798	−0.1567	0.3894	0.080*	0.560 (11)
C62A	0.17017 (8)	0.05754 (10)	0.40736 (4)	0.0297 (3)	
H62A	0.1905	0.0191	0.3920	0.036*	
O1B	0.54610 (6)	0.25191 (6)	0.45093 (2)	0.0264 (2)	
O2B	0.47966 (5)	0.40490 (7)	0.34568 (2)	0.0280 (2)	
O3B	0.23553 (5)	0.42780 (7)	0.34856 (2)	0.0264 (2)	
O4B	0.30947 (5)	0.33354 (7)	0.46325 (2)	0.0275 (2)	
N1B	0.42845 (6)	0.29231 (7)	0.45854 (3)	0.0234 (2)	
N2B	0.48873 (6)	0.31951 (7)	0.40721 (3)	0.0224 (2)	
N3B	0.35586 (6)	0.41602 (7)	0.34748 (3)	0.0234 (2)	
N4B	0.29380 (6)	0.37286 (7)	0.39651 (3)	0.0226 (2)	
C1B	0.48486 (8)	0.28997 (8)	0.43805 (3)	0.0224 (3)	
C2B	0.41791 (7)	0.39057 (9)	0.36105 (3)	0.0225 (3)	
C3B	0.29693 (7)	0.40300 (9)	0.36591 (3)	0.0222 (3)	
C4B	0.36778 (8)	0.32178 (8)	0.44505 (3)	0.0225 (3)	
C5B	0.42427 (7)	0.34930 (8)	0.39360 (3)	0.0213 (3)	
C6B	0.35961 (7)	0.34745 (8)	0.41028 (3)	0.0212 (3)	
C7B	0.55766 (8)	0.24686 (9)	0.48636 (3)	0.0248 (3)	
C8B	0.57534 (8)	0.16955 (9)	0.50036 (3)	0.0272 (3)	
H8BA	0.5748	0.1203	0.4866	0.033*	
C9B	0.59417 (8)	0.16433 (9)	0.53533 (4)	0.0280 (3)	
C10B	0.61772 (10)	0.07904 (10)	0.55111 (4)	0.0340 (3)	
C11B	0.68899 (12)	0.05144 (13)	0.53598 (5)	0.0527 (5)	
H11D	0.7264	0.0940	0.5411	0.079*	
H11E	0.6815	0.0455	0.5113	0.079*	
H11F	0.7045	−0.0028	0.5459	0.079*	
C12B	0.62986 (15)	0.08281 (12)	0.58961 (5)	0.0599 (6)	
H12D	0.5851	0.1008	0.5997	0.090*	
H12E	0.6686	0.1232	0.5956	0.090*	
H12F	0.6437	0.0268	0.5983	0.090*	
C13B	0.56126 (13)	0.01028 (12)	0.54247 (5)	0.0517 (5)	
H13D	0.5154	0.0251	0.5524	0.077*	
H13E	0.5786	−0.0439	0.5518	0.077*	
H13F	0.5537	0.0056	0.5178	0.077*	
C14B	0.59258 (8)	0.23819 (9)	0.55429 (3)	0.0279 (3)	
H14B	0.6051	0.2355	0.5779	0.033*	
C15B	0.57323 (8)	0.31656 (9)	0.53993 (3)	0.0254 (3)	
C16B	0.57356 (9)	0.39608 (10)	0.56204 (4)	0.0292 (3)	
C17B	0.65260 (10)	0.41894 (11)	0.57212 (5)	0.0425 (4)	
H17D	0.6761	0.3712	0.5840	0.064*	
H17E	0.6537	0.4683	0.5871	0.064*	
H17F	0.6783	0.4319	0.5517	0.064*	
C18B	0.53259 (12)	0.37973 (12)	0.59414 (4)	0.0456 (4)	
H18D	0.5558	0.3333	0.6070	0.068*	
H18E	0.4823	0.3646	0.5878	0.068*	
H18F	0.5335	0.4308	0.6082	0.068*	
C19B	0.53807 (10)	0.47128 (10)	0.54329 (4)	0.0362 (4)	
H19D	0.4888	0.4560	0.5351	0.054*	
H19E	0.5666	0.4866	0.5240	0.054*	
H19F	0.5360	0.5194	0.5588	0.054*	
C20B	0.55651 (8)	0.32041 (9)	0.50536 (3)	0.0248 (3)	
H20B	0.5444	0.3728	0.4948	0.030*	
C21B	0.47752 (8)	0.45907 (10)	0.31683 (3)	0.0264 (3)	
C22B	0.51408 (8)	0.53413 (10)	0.32040 (4)	0.0289 (3)	
H22B	0.5362	0.5498	0.3418	0.035*	
C23B	0.51859 (8)	0.58756 (10)	0.29228 (4)	0.0301 (3)	
C24B	0.55810 (9)	0.67259 (11)	0.29583 (4)	0.0360 (3)	0.801 (4)
C25B	0.63660 (14)	0.65958 (18)	0.30575 (10)	0.0596 (9)	0.801 (4)
H25D	0.6597	0.6287	0.2877	0.089*	0.801 (4)
H25E	0.6412	0.6271	0.3269	0.089*	0.801 (4)
H25F	0.6603	0.7145	0.3091	0.089*	0.801 (4)
C26B	0.52077 (15)	0.72795 (14)	0.32185 (6)	0.0454 (6)	0.801 (4)
H26D	0.5240	0.7003	0.3441	0.068*	0.801 (4)
H26E	0.4697	0.7358	0.3145	0.068*	0.801 (4)
H26F	0.5448	0.7830	0.3235	0.068*	0.801 (4)
C27B	0.55294 (15)	0.72480 (15)	0.26241 (6)	0.0477 (7)	0.801 (4)
H27D	0.5741	0.6924	0.2442	0.072*	0.801 (4)
H27E	0.5795	0.7779	0.2658	0.072*	0.801 (4)
H27F	0.5020	0.7369	0.2561	0.072*	0.801 (4)
C24D	0.55810 (9)	0.67259 (11)	0.29583 (4)	0.0360 (3)	0.199 (4)
C25D	0.6210 (7)	0.6598 (7)	0.3288 (3)	0.057 (3)	0.199 (4)
H25G	0.6507	0.7109	0.3313	0.086*	0.199 (4)
H25H	0.6521	0.6115	0.3243	0.086*	0.199 (4)
H25I	0.5963	0.6497	0.3498	0.086*	0.199 (4)
C26D	0.5099 (11)	0.7322 (13)	0.2997 (6)	0.105 (6)*	0.199 (4)
H26G	0.4656	0.7189	0.2860	0.157*	0.199 (4)
H26H	0.5294	0.7863	0.2923	0.157*	0.199 (4)
H26I	0.4989	0.7358	0.3237	0.157*	0.199 (4)
C27D	0.6096 (7)	0.6793 (8)	0.2668 (3)	0.064 (3)*	0.199 (4)
H27G	0.5814	0.6837	0.2452	0.097*	0.199 (4)
H27H	0.6404	0.6289	0.2666	0.097*	0.199 (4)
H27I	0.6401	0.7295	0.2701	0.097*	0.199 (4)
C28B	0.48641 (8)	0.56038 (10)	0.26153 (4)	0.0297 (3)	
H28B	0.4894	0.5956	0.2422	0.036*	
C29B	0.44983 (8)	0.48337 (10)	0.25797 (3)	0.0270 (3)	
C30B	0.41976 (9)	0.45435 (10)	0.22287 (4)	0.0319 (3)	
C31B	0.36267 (12)	0.38571 (13)	0.22561 (5)	0.0498 (5)	
H31D	0.3240	0.4061	0.2395	0.075*	
H31E	0.3850	0.3354	0.2362	0.075*	
H31F	0.3423	0.3714	0.2029	0.075*	
C32B	0.48378 (11)	0.41841 (14)	0.20413 (4)	0.0489 (5)	
H32D	0.4664	0.3977	0.1818	0.073*	
H32E	0.5059	0.3718	0.2173	0.073*	
H32F	0.5199	0.4629	0.2014	0.073*	
C33B	0.38585 (11)	0.52847 (11)	0.20266 (4)	0.0408 (4)	
H33D	0.3488	0.5553	0.2159	0.061*	
H33E	0.3636	0.5077	0.1812	0.061*	
H33F	0.4235	0.5698	0.1980	0.061*	
C34B	0.44492 (8)	0.43221 (9)	0.28637 (4)	0.0263 (3)	
H34B	0.4197	0.3800	0.2849	0.032*	
C35B	0.17072 (8)	0.43231 (10)	0.36569 (3)	0.0258 (3)	
C36B	0.11648 (8)	0.37749 (10)	0.35517 (3)	0.0276 (3)	
H36B	0.1254	0.3344	0.3392	0.033*	
C37B	0.04821 (8)	0.38584 (11)	0.36820 (4)	0.0317 (3)	
C38B	−0.01183 (9)	0.32524 (13)	0.35537 (4)	0.0398 (4)	
C39B	−0.08502 (10)	0.34739 (18)	0.36965 (6)	0.0624 (7)	
H39G	−0.0812	0.3432	0.3945	0.094*	
H39H	−0.1221	0.3080	0.3606	0.094*	
H39I	−0.0986	0.4051	0.3630	0.094*	
C40B	−0.02099 (10)	0.33130 (16)	0.31658 (4)	0.0533 (5)	
H40G	−0.0311	0.3899	0.3100	0.080*	
H40H	−0.0614	0.2953	0.3084	0.080*	
H40I	0.0236	0.3125	0.3066	0.080*	
C41B	0.00950 (10)	0.23486 (14)	0.36535 (5)	0.0501 (5)	
H41G	0.0139	0.2302	0.3901	0.075*	
H41H	0.0560	0.2209	0.3559	0.075*	
H41I	−0.0278	0.1956	0.3564	0.075*	
C42B	0.03805 (8)	0.44858 (12)	0.39217 (4)	0.0345 (3)	
H42B	−0.0084	0.4546	0.4011	0.041*	
C43B	0.09347 (9)	0.50293 (11)	0.40349 (4)	0.0325 (3)	
C44B	0.08155 (10)	0.57168 (12)	0.43013 (4)	0.0399 (4)	0.828 (4)
C45B	0.00561 (16)	0.5647 (2)	0.44420 (9)	0.0682 (10)	0.828 (4)
H45G	−0.0313	0.5735	0.4258	0.102*	0.828 (4)
H45H	0.0003	0.6076	0.4618	0.102*	0.828 (4)
H45I	−0.0005	0.5085	0.4540	0.102*	0.828 (4)
C46B	0.09038 (18)	0.65706 (15)	0.41436 (6)	0.0568 (8)	0.828 (4)
H46G	0.1399	0.6630	0.4070	0.085*	0.828 (4)
H46H	0.0809	0.7010	0.4311	0.085*	0.828 (4)
H46I	0.0560	0.6629	0.3947	0.085*	0.828 (4)
C47B	0.13748 (15)	0.56001 (16)	0.45984 (5)	0.0489 (6)	0.828 (4)
H47G	0.1863	0.5669	0.4517	0.073*	0.828 (4)
H47H	0.1326	0.5034	0.4694	0.073*	0.828 (4)
H47I	0.1293	0.6023	0.4773	0.073*	0.828 (4)
C44D	0.08155 (10)	0.57168 (12)	0.43013 (4)	0.0399 (4)	0.172 (4)
C45D	0.0218 (10)	0.6420 (9)	0.4154 (4)	0.073 (5)	0.172 (4)
H45J	−0.0208	0.6129	0.4053	0.109*	0.172 (4)
H45K	0.0436	0.6769	0.3981	0.109*	0.172 (4)
H45L	0.0073	0.6780	0.4340	0.109*	0.172 (4)
C46D	0.1501 (9)	0.6337 (12)	0.4395 (4)	0.085 (6)	0.172 (4)
H46J	0.1904	0.6005	0.4496	0.127*	0.172 (4)
H46K	0.1358	0.6767	0.4557	0.127*	0.172 (4)
H46L	0.1652	0.6614	0.4188	0.127*	0.172 (4)
C47D	0.0567 (13)	0.5434 (10)	0.4624 (4)	0.080 (6)	0.172 (4)
H47J	0.0920	0.5038	0.4728	0.120*	0.172 (4)
H47K	0.0098	0.5150	0.4587	0.120*	0.172 (4)
H47L	0.0514	0.5920	0.4774	0.120*	0.172 (4)
C48B	0.16104 (8)	0.49488 (10)	0.38948 (4)	0.0294 (3)	
H48B	0.1997	0.5318	0.3962	0.035*	
C49B	0.31608 (8)	0.32787 (10)	0.49899 (3)	0.0270 (3)	
C50B	0.32876 (8)	0.25091 (10)	0.51467 (4)	0.0290 (3)	
H50B	0.3380	0.2019	0.5017	0.035*	
C51B	0.32764 (8)	0.24702 (10)	0.54994 (4)	0.0313 (3)	
C52B	0.33858 (10)	0.16366 (11)	0.56920 (4)	0.0385 (4)	
C53B	0.26949 (13)	0.14339 (15)	0.58758 (5)	0.0589 (6)	
H53D	0.2283	0.1394	0.5710	0.088*	
H53E	0.2605	0.1883	0.6039	0.088*	
H53F	0.2756	0.0895	0.5996	0.088*	
C54B	0.40305 (13)	0.17163 (14)	0.59514 (5)	0.0558 (5)	
H54D	0.4469	0.1850	0.5833	0.084*	
H54E	0.4099	0.1182	0.6074	0.084*	
H54F	0.3935	0.2168	0.6113	0.084*	
C55B	0.35358 (13)	0.09045 (12)	0.54540 (5)	0.0527 (5)	
H55D	0.3976	0.1022	0.5335	0.079*	
H55E	0.3126	0.0837	0.5288	0.079*	
H55F	0.3601	0.0384	0.5586	0.079*	
C56B	0.31424 (9)	0.32095 (11)	0.56768 (4)	0.0351 (3)	
H56B	0.3139	0.3184	0.5917	0.042*	
C57B	0.30132 (9)	0.39841 (11)	0.55181 (4)	0.0340 (3)	
C58B	0.28413 (11)	0.47920 (12)	0.57138 (4)	0.0438 (4)	0.809 (4)
C59B	0.2886 (3)	0.4655 (2)	0.60968 (7)	0.0890 (15)	0.809 (4)
H59G	0.3376	0.4471	0.6170	0.134*	0.809 (4)
H59H	0.2537	0.4220	0.6156	0.134*	0.809 (4)
H59I	0.2774	0.5184	0.6211	0.134*	0.809 (4)
C60B	0.20934 (16)	0.5100 (2)	0.55992 (9)	0.0770 (12)	0.809 (4)
H60G	0.2076	0.5211	0.5355	0.116*	0.809 (4)
H60H	0.1984	0.5620	0.5721	0.116*	0.809 (4)
H60I	0.1735	0.4667	0.5648	0.116*	0.809 (4)
C61B	0.33951 (17)	0.54803 (17)	0.56392 (8)	0.0625 (9)	0.809 (4)
H61G	0.3885	0.5273	0.5699	0.094*	0.809 (4)
H61H	0.3300	0.5984	0.5774	0.094*	0.809 (4)
H61I	0.3354	0.5623	0.5397	0.094*	0.809 (4)
C58D	0.28413 (11)	0.47920 (12)	0.57138 (4)	0.0438 (4)	0.191 (4)
C59D	0.2228 (8)	0.4572 (10)	0.5964 (4)	0.072 (4)*	0.191 (4)
H59J	0.2121	0.5073	0.6099	0.107*	0.191 (4)
H59K	0.2395	0.4115	0.6116	0.107*	0.191 (4)
H59L	0.1790	0.4394	0.5833	0.107*	0.191 (4)
C61D	0.3518 (9)	0.5047 (11)	0.5946 (4)	0.086 (5)*	0.191 (4)
H61J	0.3381	0.5487	0.6105	0.129*	0.191 (4)
H61K	0.3898	0.5261	0.5806	0.129*	0.191 (4)
H61L	0.3697	0.4552	0.6074	0.129*	0.191 (4)
C60D	0.2627 (12)	0.5522 (13)	0.5494 (5)	0.106 (6)*	0.191 (4)
H60J	0.2485	0.5995	0.5636	0.159*	0.191 (4)
H60K	0.2217	0.5360	0.5340	0.159*	0.191 (4)
H60L	0.3036	0.5691	0.5362	0.159*	0.191 (4)
C62B	0.30251 (8)	0.40060 (10)	0.51637 (4)	0.0310 (3)	
H62B	0.2940	0.4521	0.5045	0.037*	

### Atomic displacement parameters (Å^2^)

**Table:**

	*U*^11^	*U*^22^	*U*^33^	*U*^12^	*U*^13^	*U*^23^
O1A	0.0192 (4)	0.0344 (5)	0.0196 (4)	0.0024 (4)	−0.0008 (3)	0.0048 (4)
O2A	0.0191 (4)	0.0324 (5)	0.0197 (4)	0.0004 (4)	0.0020 (3)	0.0058 (4)
O3A	0.0187 (5)	0.0358 (5)	0.0208 (4)	0.0023 (4)	0.0003 (3)	0.0053 (4)
O4A	0.0188 (4)	0.0373 (5)	0.0184 (4)	−0.0003 (4)	0.0015 (3)	0.0046 (4)
N1A	0.0200 (5)	0.0250 (5)	0.0199 (5)	−0.0001 (4)	0.0005 (4)	0.0012 (4)
N2A	0.0200 (5)	0.0262 (5)	0.0196 (5)	0.0003 (4)	0.0000 (4)	0.0024 (4)
N3A	0.0199 (5)	0.0231 (5)	0.0201 (5)	−0.0011 (4)	0.0003 (4)	0.0004 (4)
N4A	0.0196 (5)	0.0223 (5)	0.0185 (5)	−0.0009 (4)	−0.0009 (4)	0.0005 (4)
C1A	0.0209 (6)	0.0230 (6)	0.0193 (6)	0.0009 (5)	−0.0019 (5)	0.0006 (5)
C2A	0.0213 (6)	0.0212 (6)	0.0189 (6)	−0.0012 (5)	0.0000 (5)	0.0010 (5)
C3A	0.0201 (6)	0.0197 (6)	0.0185 (6)	−0.0021 (5)	−0.0025 (4)	0.0004 (5)
C4A	0.0225 (6)	0.0209 (6)	0.0183 (6)	−0.0018 (5)	0.0012 (5)	0.0000 (5)
C5A	0.0200 (6)	0.0209 (6)	0.0187 (6)	−0.0014 (5)	−0.0005 (5)	0.0000 (5)
C6A	0.0205 (6)	0.0194 (6)	0.0189 (6)	−0.0016 (5)	−0.0001 (5)	−0.0008 (5)
C7A	0.0192 (6)	0.0297 (7)	0.0188 (6)	−0.0007 (5)	−0.0028 (5)	0.0037 (5)
C8A	0.0244 (7)	0.0283 (7)	0.0223 (6)	0.0020 (5)	−0.0009 (5)	−0.0022 (5)
C9A	0.0260 (7)	0.0248 (7)	0.0244 (6)	0.0007 (5)	−0.0030 (5)	0.0011 (5)
C10A	0.0402 (9)	0.0275 (7)	0.0283 (7)	0.0086 (6)	−0.0030 (6)	0.0019 (6)
C11A	0.0744 (16)	0.0656 (14)	0.0768 (15)	0.0464 (13)	0.0305 (13)	0.0304 (12)
C12A	0.0777 (14)	0.0319 (8)	0.0363 (9)	0.0172 (9)	−0.0194 (9)	0.0024 (7)
C13A	0.0721 (14)	0.0250 (8)	0.0666 (13)	0.0053 (9)	−0.0294 (11)	−0.0009 (8)
C14A	0.0273 (7)	0.0269 (7)	0.0199 (6)	−0.0001 (6)	−0.0030 (5)	0.0022 (5)
C15A	0.0218 (6)	0.0256 (6)	0.0218 (6)	−0.0017 (5)	−0.0029 (5)	−0.0012 (5)
C16A	0.0291 (7)	0.0287 (7)	0.0231 (6)	0.0024 (6)	−0.0010 (5)	−0.0026 (5)
C17A	0.0415 (9)	0.0364 (8)	0.0449 (9)	−0.0045 (7)	0.0067 (7)	−0.0138 (7)
C18A	0.0529 (10)	0.0438 (9)	0.0229 (7)	0.0091 (8)	0.0015 (7)	−0.0028 (6)
C19A	0.0337 (8)	0.0461 (9)	0.0304 (7)	0.0089 (7)	0.0006 (6)	−0.0047 (7)
C20A	0.0202 (6)	0.0236 (6)	0.0249 (6)	0.0010 (5)	−0.0023 (5)	0.0019 (5)
C21A	0.0202 (6)	0.0304 (7)	0.0226 (6)	0.0019 (5)	0.0033 (5)	0.0084 (5)
C22A	0.0254 (7)	0.0313 (7)	0.0232 (6)	0.0018 (6)	0.0017 (5)	0.0065 (5)
C23A	0.0295 (7)	0.0338 (7)	0.0255 (7)	0.0060 (6)	0.0057 (5)	0.0080 (6)
C24A	0.0367 (8)	0.0451 (9)	0.0286 (7)	0.0077 (7)	0.0109 (6)	0.0036 (7)
C25A	0.0561 (12)	0.0646 (13)	0.0496 (11)	0.0190 (11)	0.0088 (9)	−0.0082 (10)
C26A	0.0516 (12)	0.0915 (17)	0.0366 (9)	−0.0008 (12)	0.0093 (8)	−0.0186 (10)
C27A	0.105 (2)	0.0616 (13)	0.0338 (9)	−0.0009 (13)	0.0267 (11)	0.0055 (9)
C28A	0.0227 (7)	0.0376 (8)	0.0325 (7)	0.0047 (6)	0.0087 (5)	0.0123 (6)
C29A	0.0216 (7)	0.0321 (7)	0.0304 (7)	0.0011 (6)	0.0020 (5)	0.0116 (6)
C30A	0.0209 (7)	0.0411 (8)	0.0387 (8)	−0.0049 (6)	0.0014 (6)	0.0100 (7)
C31A	0.0216 (8)	0.0693 (13)	0.0502 (10)	−0.0040 (8)	0.0030 (7)	0.0121 (9)
C32A	0.0321 (9)	0.0405 (10)	0.0743 (13)	−0.0092 (8)	−0.0012 (8)	0.0089 (9)
C33A	0.0330 (8)	0.0599 (11)	0.0364 (8)	−0.0121 (8)	−0.0039 (7)	0.0067 (8)
C34A	0.0224 (7)	0.0304 (7)	0.0241 (6)	0.0003 (5)	0.0025 (5)	0.0073 (5)
C35A	0.0196 (6)	0.0249 (6)	0.0250 (6)	0.0011 (5)	0.0014 (5)	0.0041 (5)
C36A	0.0255 (7)	0.0269 (7)	0.0274 (7)	−0.0006 (6)	0.0011 (5)	−0.0007 (5)
C37A	0.0304 (8)	0.0287 (7)	0.0370 (8)	0.0014 (6)	0.0093 (6)	−0.0030 (6)
C38A	0.0381 (9)	0.0471 (10)	0.0438 (9)	0.0079 (8)	0.0105 (7)	−0.0123 (8)
C39A	0.061 (2)	0.0478 (18)	0.0343 (15)	0.0134 (16)	0.0040 (13)	−0.0112 (13)
C40A	0.058 (2)	0.0455 (17)	0.0446 (17)	0.0104 (15)	0.0247 (15)	0.0074 (14)
C41A	0.064 (2)	0.0401 (17)	0.0398 (16)	0.0239 (17)	0.0077 (15)	0.0015 (13)
C38C	0.0381 (9)	0.0471 (10)	0.0438 (9)	0.0079 (8)	0.0105 (7)	−0.0123 (8)
C39C	0.062 (3)	0.035 (2)	0.0290 (19)	−0.0020 (19)	0.0084 (18)	−0.0070 (16)
C40C	0.054 (3)	0.042 (2)	0.0268 (18)	0.014 (2)	0.0110 (17)	0.0084 (16)
C41C	0.042 (3)	0.060 (3)	0.046 (2)	0.017 (2)	0.010 (2)	−0.010 (2)
C42A	0.0206 (7)	0.0323 (8)	0.0490 (9)	0.0008 (6)	0.0076 (6)	−0.0018 (7)
C43A	0.0218 (7)	0.0260 (7)	0.0401 (8)	−0.0017 (6)	−0.0024 (6)	0.0008 (6)
C44A	0.0236 (7)	0.0371 (8)	0.0495 (9)	−0.0051 (6)	−0.0061 (7)	−0.0022 (7)
C45A	0.044 (2)	0.072 (3)	0.048 (2)	−0.035 (2)	−0.0093 (17)	0.014 (2)
C46A	0.0403 (19)	0.045 (2)	0.0408 (18)	−0.0052 (16)	−0.0136 (15)	−0.0108 (15)
C47A	0.0304 (17)	0.053 (2)	0.0352 (17)	0.0026 (15)	−0.0093 (13)	0.0041 (15)
C44C	0.0236 (7)	0.0371 (8)	0.0495 (9)	−0.0051 (6)	−0.0061 (7)	−0.0022 (7)
C45C	0.0233 (17)	0.057 (2)	0.096 (3)	0.0034 (16)	−0.0156 (18)	−0.027 (2)
C46C	0.0321 (16)	0.0397 (18)	0.0466 (18)	−0.0060 (14)	−0.0019 (13)	−0.0104 (14)
C47C	0.050 (2)	0.060 (2)	0.050 (2)	−0.0133 (19)	−0.0296 (18)	0.0114 (18)
C48A	0.0231 (7)	0.0247 (6)	0.0279 (7)	0.0003 (5)	0.0002 (5)	0.0005 (5)
C49A	0.0192 (6)	0.0340 (7)	0.0192 (6)	−0.0009 (5)	0.0015 (5)	0.0056 (5)
C50A	0.0228 (7)	0.0327 (7)	0.0211 (6)	−0.0031 (6)	−0.0005 (5)	0.0010 (5)
C51A	0.0240 (7)	0.0372 (8)	0.0218 (6)	−0.0019 (6)	0.0019 (5)	−0.0003 (6)
C52A	0.0249 (7)	0.0438 (9)	0.0260 (7)	−0.0043 (6)	0.0042 (5)	−0.0067 (6)
C53A	0.0278 (9)	0.0831 (15)	0.0562 (11)	−0.0023 (9)	0.0073 (8)	−0.0369 (11)
C54A	0.0572 (12)	0.0418 (10)	0.0525 (11)	0.0006 (9)	0.0227 (9)	−0.0101 (8)
C55A	0.0609 (12)	0.0663 (13)	0.0252 (8)	0.0023 (10)	0.0004 (8)	−0.0078 (8)
C56A	0.0293 (8)	0.0431 (9)	0.0347 (8)	−0.0116 (7)	0.0132 (6)	−0.0053 (7)
C57A	0.0370 (9)	0.0385 (8)	0.0381 (8)	−0.0128 (7)	0.0136 (7)	−0.0073 (7)
C58A	0.0725 (16)	0.0599 (13)	0.0948 (18)	−0.0411 (12)	0.0554 (14)	−0.0412 (13)
C59A	0.074 (4)	0.031 (2)	0.047 (3)	−0.011 (2)	0.031 (3)	0.0011 (19)
C60A	0.036 (2)	0.042 (3)	0.095 (6)	−0.007 (2)	−0.005 (3)	−0.015 (3)
C61A	0.0682 (14)	0.0398 (10)	0.0534 (11)	−0.0202 (10)	0.0210 (10)	−0.0132 (8)
C58C	0.0725 (16)	0.0599 (13)	0.0948 (18)	−0.0411 (12)	0.0554 (14)	−0.0412 (13)
C59C	0.102 (6)	0.054 (3)	0.065 (3)	−0.038 (3)	0.039 (4)	−0.006 (3)
C60C	0.038 (2)	0.064 (3)	0.078 (4)	−0.020 (2)	0.002 (2)	−0.029 (3)
C61C	0.0682 (14)	0.0398 (10)	0.0534 (11)	−0.0202 (10)	0.0210 (10)	−0.0132 (8)
C62A	0.0298 (7)	0.0328 (7)	0.0270 (7)	−0.0024 (6)	0.0067 (6)	−0.0018 (6)
O1B	0.0300 (5)	0.0315 (5)	0.0174 (4)	0.0078 (4)	−0.0022 (4)	0.0010 (4)
O2B	0.0225 (5)	0.0396 (6)	0.0220 (4)	0.0039 (4)	0.0023 (4)	0.0100 (4)
O3B	0.0202 (5)	0.0388 (5)	0.0201 (4)	0.0018 (4)	0.0004 (3)	0.0034 (4)
O4B	0.0247 (5)	0.0406 (6)	0.0171 (4)	−0.0021 (4)	0.0007 (4)	0.0020 (4)
N1B	0.0269 (6)	0.0248 (5)	0.0181 (5)	−0.0022 (5)	−0.0018 (4)	0.0001 (4)
N2B	0.0243 (6)	0.0236 (5)	0.0191 (5)	0.0014 (4)	−0.0014 (4)	0.0002 (4)
N3B	0.0223 (6)	0.0273 (6)	0.0203 (5)	0.0006 (5)	−0.0001 (4)	0.0017 (4)
N4B	0.0224 (5)	0.0254 (5)	0.0199 (5)	−0.0028 (4)	−0.0008 (4)	−0.0012 (4)
C1B	0.0256 (7)	0.0219 (6)	0.0193 (6)	0.0010 (5)	−0.0032 (5)	−0.0022 (5)
C2B	0.0232 (6)	0.0247 (6)	0.0193 (6)	−0.0002 (5)	0.0002 (5)	−0.0001 (5)
C3B	0.0222 (6)	0.0243 (6)	0.0196 (6)	−0.0006 (5)	−0.0021 (5)	−0.0017 (5)
C4B	0.0255 (7)	0.0220 (6)	0.0198 (6)	−0.0048 (5)	0.0002 (5)	−0.0013 (5)
C5B	0.0247 (7)	0.0204 (6)	0.0185 (6)	−0.0010 (5)	−0.0016 (5)	−0.0014 (5)
C6B	0.0247 (6)	0.0204 (6)	0.0182 (6)	−0.0039 (5)	−0.0008 (5)	−0.0014 (5)
C7B	0.0251 (7)	0.0309 (7)	0.0181 (6)	0.0005 (6)	−0.0016 (5)	0.0009 (5)
C8B	0.0316 (7)	0.0275 (7)	0.0220 (6)	0.0004 (6)	−0.0037 (5)	−0.0019 (5)
C9B	0.0331 (7)	0.0284 (7)	0.0219 (6)	−0.0017 (6)	−0.0042 (5)	0.0025 (5)
C10B	0.0480 (9)	0.0281 (7)	0.0246 (7)	−0.0015 (7)	−0.0104 (6)	0.0042 (6)
C11B	0.0601 (13)	0.0423 (10)	0.0544 (11)	0.0126 (9)	−0.0084 (9)	0.0115 (9)
C12B	0.113 (2)	0.0365 (9)	0.0284 (8)	0.0043 (11)	−0.0168 (10)	0.0054 (7)
C13B	0.0706 (14)	0.0353 (9)	0.0469 (10)	−0.0112 (9)	−0.0171 (9)	0.0109 (8)
C14B	0.0327 (7)	0.0312 (7)	0.0189 (6)	−0.0036 (6)	−0.0053 (5)	0.0015 (5)
C15B	0.0254 (7)	0.0281 (7)	0.0224 (6)	−0.0043 (5)	−0.0019 (5)	−0.0009 (5)
C16B	0.0360 (8)	0.0288 (7)	0.0220 (6)	−0.0037 (6)	−0.0050 (6)	−0.0031 (5)
C17B	0.0438 (10)	0.0345 (8)	0.0469 (9)	−0.0060 (7)	−0.0170 (8)	−0.0041 (7)
C18B	0.0672 (13)	0.0397 (9)	0.0307 (8)	−0.0019 (9)	0.0105 (8)	−0.0054 (7)
C19B	0.0479 (10)	0.0295 (7)	0.0302 (7)	0.0019 (7)	−0.0089 (7)	−0.0060 (6)
C20B	0.0254 (7)	0.0264 (7)	0.0224 (6)	−0.0001 (5)	−0.0011 (5)	0.0015 (5)
C21B	0.0218 (6)	0.0354 (7)	0.0222 (6)	0.0046 (6)	0.0038 (5)	0.0080 (6)
C22B	0.0214 (6)	0.0429 (8)	0.0223 (6)	−0.0015 (6)	0.0003 (5)	0.0020 (6)
C23B	0.0255 (7)	0.0372 (8)	0.0279 (7)	−0.0022 (6)	0.0035 (5)	0.0041 (6)
C24B	0.0321 (8)	0.0429 (9)	0.0329 (8)	−0.0100 (7)	0.0012 (6)	0.0028 (7)
C25B	0.0313 (12)	0.0498 (15)	0.095 (3)	−0.0092 (11)	−0.0182 (13)	0.0156 (15)
C26B	0.0592 (15)	0.0327 (11)	0.0455 (13)	−0.0127 (10)	0.0139 (11)	−0.0015 (9)
C27B	0.0635 (16)	0.0442 (13)	0.0349 (11)	−0.0216 (12)	−0.0013 (10)	0.0106 (9)
C24D	0.0321 (8)	0.0429 (9)	0.0329 (8)	−0.0100 (7)	0.0012 (6)	0.0028 (7)
C25D	0.076 (8)	0.063 (7)	0.032 (5)	−0.039 (6)	−0.003 (5)	0.005 (5)
C28B	0.0305 (7)	0.0342 (7)	0.0245 (7)	−0.0007 (6)	0.0023 (5)	0.0076 (6)
C29B	0.0256 (7)	0.0328 (7)	0.0224 (6)	0.0029 (6)	−0.0002 (5)	0.0039 (6)
C30B	0.0393 (8)	0.0325 (8)	0.0233 (7)	0.0038 (7)	−0.0046 (6)	0.0040 (6)
C31B	0.0624 (12)	0.0484 (10)	0.0364 (9)	−0.0138 (9)	−0.0195 (8)	0.0053 (8)
C32B	0.0573 (12)	0.0579 (11)	0.0308 (8)	0.0199 (10)	−0.0040 (8)	−0.0060 (8)
C33B	0.0558 (11)	0.0369 (8)	0.0282 (7)	0.0086 (8)	−0.0124 (7)	0.0027 (6)
C34B	0.0235 (7)	0.0294 (7)	0.0262 (7)	0.0029 (6)	0.0017 (5)	0.0048 (6)
C35B	0.0217 (6)	0.0353 (7)	0.0205 (6)	0.0035 (6)	0.0008 (5)	0.0049 (5)
C36B	0.0240 (7)	0.0381 (8)	0.0206 (6)	0.0022 (6)	−0.0006 (5)	0.0002 (6)
C37B	0.0225 (7)	0.0486 (9)	0.0238 (7)	−0.0001 (6)	−0.0008 (5)	−0.0001 (6)
C38B	0.0222 (7)	0.0656 (11)	0.0318 (8)	−0.0053 (7)	0.0024 (6)	−0.0080 (8)
C39B	0.0234 (8)	0.1078 (19)	0.0563 (12)	−0.0105 (10)	0.0054 (8)	−0.0287 (12)
C40B	0.0349 (9)	0.0915 (16)	0.0328 (9)	−0.0118 (10)	−0.0043 (7)	−0.0106 (9)
C41B	0.0361 (9)	0.0612 (12)	0.0532 (11)	−0.0174 (9)	0.0049 (8)	−0.0025 (9)
C42B	0.0236 (7)	0.0535 (10)	0.0266 (7)	0.0049 (7)	0.0035 (5)	−0.0013 (7)
C43B	0.0320 (8)	0.0413 (8)	0.0241 (7)	0.0057 (7)	0.0015 (6)	−0.0014 (6)
C44B	0.0398 (9)	0.0464 (10)	0.0340 (8)	0.0030 (8)	0.0068 (7)	−0.0089 (7)
C45B	0.0513 (16)	0.084 (2)	0.072 (2)	−0.0082 (15)	0.0264 (14)	−0.0470 (18)
C46B	0.086 (2)	0.0396 (12)	0.0457 (13)	0.0121 (13)	0.0065 (13)	−0.0071 (10)
C47B	0.0629 (15)	0.0530 (14)	0.0302 (10)	0.0036 (12)	−0.0019 (10)	−0.0131 (9)
C44D	0.0398 (9)	0.0464 (10)	0.0340 (8)	0.0030 (8)	0.0068 (7)	−0.0089 (7)
C45D	0.090 (12)	0.055 (8)	0.073 (9)	0.026 (8)	−0.005 (8)	−0.015 (7)
C46D	0.077 (11)	0.099 (13)	0.079 (11)	−0.018 (9)	0.013 (8)	−0.051 (10)
C47D	0.133 (18)	0.056 (8)	0.054 (8)	−0.004 (10)	0.043 (10)	−0.015 (7)
C48B	0.0288 (7)	0.0349 (8)	0.0244 (6)	0.0004 (6)	−0.0004 (5)	0.0012 (6)
C49B	0.0235 (7)	0.0395 (8)	0.0180 (6)	−0.0053 (6)	0.0006 (5)	0.0013 (5)
C50B	0.0292 (7)	0.0356 (8)	0.0222 (7)	−0.0064 (6)	0.0002 (5)	0.0000 (6)
C51B	0.0322 (8)	0.0389 (8)	0.0225 (7)	−0.0098 (6)	−0.0007 (5)	0.0043 (6)
C52B	0.0485 (10)	0.0412 (9)	0.0251 (7)	−0.0111 (8)	−0.0033 (7)	0.0080 (6)
C53B	0.0665 (14)	0.0653 (13)	0.0455 (10)	−0.0150 (11)	0.0099 (10)	0.0243 (10)
C54B	0.0679 (14)	0.0505 (11)	0.0463 (10)	−0.0078 (10)	−0.0224 (10)	0.0137 (9)
C55B	0.0824 (15)	0.0367 (9)	0.0387 (9)	−0.0056 (10)	−0.0010 (9)	0.0095 (8)
C56B	0.0397 (8)	0.0479 (9)	0.0179 (6)	−0.0088 (7)	0.0016 (6)	0.0002 (6)
C57B	0.0363 (8)	0.0420 (9)	0.0239 (7)	−0.0054 (7)	0.0025 (6)	−0.0029 (6)
C58B	0.0535 (11)	0.0476 (10)	0.0307 (8)	−0.0003 (9)	0.0072 (7)	−0.0082 (7)
C59B	0.172 (5)	0.0635 (19)	0.0328 (13)	0.003 (2)	0.0167 (18)	−0.0167 (13)
C60B	0.0482 (16)	0.092 (2)	0.091 (2)	0.0112 (16)	0.0064 (15)	−0.053 (2)
C61B	0.0620 (17)	0.0447 (14)	0.082 (2)	−0.0083 (12)	0.0168 (15)	−0.0245 (14)
C58D	0.0535 (11)	0.0476 (10)	0.0307 (8)	−0.0003 (9)	0.0072 (7)	−0.0082 (7)
C62B	0.0321 (8)	0.0360 (8)	0.0250 (7)	−0.0029 (6)	0.0026 (6)	0.0018 (6)

### Geometric parameters (Å, º)

**Table:**

O1A—C1A	1.3557 (15)	O1B—C1B	1.3572 (16)
O1A—C7A	1.4071 (15)	O1B—C7B	1.4053 (15)
O2A—C2A	1.3417 (15)	O2B—C2B	1.3404 (16)
O2A—C21A	1.4110 (15)	O2B—C21B	1.4247 (16)
O3A—C3A	1.3467 (15)	O3B—C3B	1.3520 (16)
O3A—C35A	1.4005 (16)	O3B—C35B	1.4086 (16)
O4A—C4A	1.3355 (15)	O4B—C4B	1.3398 (17)
O4A—C49A	1.4128 (15)	O4B—C49B	1.4121 (15)
N1A—C4A	1.3025 (17)	N1B—C4B	1.3021 (18)
N1A—C1A	1.3638 (16)	N1B—C1B	1.3537 (18)
N2A—C1A	1.2988 (17)	N2B—C1B	1.3095 (17)
N2A—C5A	1.3685 (16)	N2B—C5B	1.3649 (17)
N3A—C2A	1.3101 (17)	N3B—C2B	1.3037 (18)
N3A—C3A	1.3537 (16)	N3B—C3B	1.3574 (17)
N4A—C3A	1.3104 (17)	N4B—C3B	1.3027 (17)
N4A—C6A	1.3579 (16)	N4B—C6B	1.3662 (17)
C2A—C5A	1.4300 (18)	C2B—C5B	1.4404 (17)
C4A—C6A	1.4399 (18)	C4B—C6B	1.4313 (17)
C5A—C6A	1.3907 (17)	C5B—C6B	1.3954 (18)
C7A—C20A	1.3803 (19)	C7B—C8B	1.376 (2)
C7A—C8A	1.3808 (19)	C7B—C20B	1.386 (2)
C8A—C9A	1.3916 (19)	C8B—C9B	1.4076 (18)
C8A—H8AA	0.9500	C8B—H8BA	0.9500
C9A—C14A	1.393 (2)	C9B—C14B	1.390 (2)
C9A—C10A	1.5319 (19)	C9B—C10B	1.541 (2)
C10A—C13A	1.521 (3)	C10B—C12B	1.525 (2)
C10A—C11A	1.525 (3)	C10B—C13B	1.534 (2)
C10A—C12A	1.526 (2)	C10B—C11B	1.539 (3)
C11A—H11A	0.9800	C11B—H11D	0.9800
C11A—H11B	0.9800	C11B—H11E	0.9800
C11A—H11C	0.9800	C11B—H11F	0.9800
C12A—H12A	0.9800	C12B—H12D	0.9800
C12A—H12B	0.9800	C12B—H12E	0.9800
C12A—H12C	0.9800	C12B—H12F	0.9800
C13A—H13A	0.9800	C13B—H13D	0.9800
C13A—H13B	0.9800	C13B—H13E	0.9800
C13A—H13C	0.9800	C13B—H13F	0.9800
C14A—C15A	1.3951 (19)	C14B—C15B	1.403 (2)
C14A—H14A	0.9500	C14B—H14B	0.9500
C15A—C20A	1.3982 (18)	C15B—C20B	1.3838 (18)
C15A—C16A	1.5293 (18)	C15B—C16B	1.5325 (19)
C16A—C17A	1.525 (2)	C16B—C19B	1.532 (2)
C16A—C19A	1.535 (2)	C16B—C18B	1.532 (2)
C16A—C18A	1.537 (2)	C16B—C17B	1.537 (2)
C17A—H17A	0.9800	C17B—H17D	0.9800
C17A—H17B	0.9800	C17B—H17E	0.9800
C17A—H17C	0.9800	C17B—H17F	0.9800
C18A—H18A	0.9800	C18B—H18D	0.9800
C18A—H18B	0.9800	C18B—H18E	0.9800
C18A—H18C	0.9800	C18B—H18F	0.9800
C19A—H19A	0.9800	C19B—H19D	0.9800
C19A—H19B	0.9800	C19B—H19E	0.9800
C19A—H19C	0.9800	C19B—H19F	0.9800
C20A—H20A	0.9500	C20B—H20B	0.9500
C21A—C34A	1.378 (2)	C21B—C22B	1.371 (2)
C21A—C22A	1.384 (2)	C21B—C34B	1.382 (2)
C22A—C23A	1.396 (2)	C22B—C23B	1.402 (2)
C22A—H22A	0.9500	C22B—H22B	0.9500
C23A—C28A	1.401 (2)	C23B—C28B	1.391 (2)
C23A—C24A	1.539 (2)	C23B—C24B	1.535 (2)
C24A—C27A	1.520 (3)	C23B—C24D	1.535 (2)
C24A—C26A	1.530 (3)	C24B—C25B	1.497 (3)
C24A—C25A	1.534 (3)	C24B—C26B	1.541 (3)
C25A—H25A	0.9800	C24B—C27B	1.555 (3)
C25A—H25B	0.9800	C25B—H25D	0.9800
C25A—H25C	0.9800	C25B—H25E	0.9800
C26A—H26A	0.9800	C25B—H25F	0.9800
C26A—H26B	0.9800	C26B—H26D	0.9800
C26A—H26C	0.9800	C26B—H26E	0.9800
C27A—H27A	0.9800	C26B—H26F	0.9800
C27A—H27B	0.9800	C27B—H27D	0.9800
C27A—H27C	0.9800	C27B—H27E	0.9800
C28A—C29A	1.394 (2)	C27B—H27F	0.9800
C28A—H28A	0.9500	C24D—C26D	1.31 (2)
C29A—C34A	1.4004 (19)	C24D—C27D	1.532 (12)
C29A—C30A	1.536 (2)	C24D—C25D	1.711 (11)
C30A—C31A	1.531 (2)	C25D—H25G	0.9800
C30A—C32A	1.537 (2)	C25D—H25H	0.9800
C30A—C33A	1.538 (2)	C25D—H25I	0.9800
C31A—H31A	0.9800	C26D—H26G	0.9800
C31A—H31B	0.9800	C26D—H26H	0.9800
C31A—H31C	0.9800	C26D—H26I	0.9800
C32A—H32A	0.9800	C27D—H27G	0.9800
C32A—H32B	0.9800	C27D—H27H	0.9800
C32A—H32C	0.9800	C27D—H27I	0.9800
C33A—H33A	0.9800	C28B—C29B	1.398 (2)
C33A—H33B	0.9800	C28B—H28B	0.9500
C33A—H33C	0.9800	C29B—C34B	1.3902 (19)
C34A—H34A	0.9500	C29B—C30B	1.5351 (19)
C35A—C48A	1.3812 (19)	C30B—C31B	1.523 (2)
C35A—C36A	1.3849 (19)	C30B—C33B	1.534 (2)
C36A—C37A	1.392 (2)	C30B—C32B	1.538 (2)
C36A—H36A	0.9500	C31B—H31D	0.9800
C37A—C42A	1.398 (2)	C31B—H31E	0.9800
C37A—C38A	1.535 (2)	C31B—H31F	0.9800
C37A—C38C	1.535 (2)	C32B—H32D	0.9800
C38A—C41A	1.432 (3)	C32B—H32E	0.9800
C38A—C39A	1.577 (4)	C32B—H32F	0.9800
C38A—C40A	1.637 (4)	C33B—H33D	0.9800
C39A—H39A	0.9800	C33B—H33E	0.9800
C39A—H39B	0.9800	C33B—H33F	0.9800
C39A—H39C	0.9800	C34B—H34B	0.9500
C40A—H40A	0.9800	C35B—C36B	1.374 (2)
C40A—H40B	0.9800	C35B—C48B	1.383 (2)
C40A—H40C	0.9800	C36B—C37B	1.394 (2)
C41A—H41A	0.9800	C36B—H36B	0.9500
C41A—H41B	0.9800	C37B—C42B	1.392 (2)
C41A—H41C	0.9800	C37B—C38B	1.533 (2)
C38C—C40C	1.347 (4)	C38B—C41B	1.531 (3)
C38C—C41C	1.591 (5)	C38B—C40B	1.534 (2)
C38C—C39C	1.662 (5)	C38B—C39B	1.534 (2)
C39C—H39D	0.9800	C39B—H39G	0.9800
C39C—H39E	0.9800	C39B—H39H	0.9800
C39C—H39F	0.9800	C39B—H39I	0.9800
C40C—H40D	0.9800	C40B—H40G	0.9800
C40C—H40E	0.9800	C40B—H40H	0.9800
C40C—H40F	0.9800	C40B—H40I	0.9800
C41C—H41D	0.9800	C41B—H41G	0.9800
C41C—H41E	0.9800	C41B—H41H	0.9800
C41C—H41F	0.9800	C41B—H41I	0.9800
C42A—C43A	1.394 (2)	C42B—C43B	1.394 (2)
C42A—H42A	0.9500	C42B—H42B	0.9500
C43A—C48A	1.393 (2)	C43B—C48B	1.399 (2)
C43A—C44A	1.535 (2)	C43B—C44B	1.538 (2)
C43A—C44C	1.535 (2)	C43B—C44D	1.538 (2)
C44A—C45A	1.471 (4)	C44B—C46B	1.502 (3)
C44A—C46A	1.582 (4)	C44B—C47B	1.532 (3)
C44A—C47A	1.605 (4)	C44B—C45B	1.541 (3)
C45A—H45A	0.9800	C45B—H45G	0.9800
C45A—H45B	0.9800	C45B—H45H	0.9800
C45A—H45C	0.9800	C45B—H45I	0.9800
C46A—H46A	0.9800	C46B—H46G	0.9800
C46A—H46B	0.9800	C46B—H46H	0.9800
C46A—H46C	0.9800	C46B—H46I	0.9800
C47A—H47A	0.9800	C47B—H47G	0.9800
C47A—H47B	0.9800	C47B—H47H	0.9800
C47A—H47C	0.9800	C47B—H47I	0.9800
C44C—C47C	1.432 (4)	C44D—C47D	1.446 (14)
C44C—C45C	1.551 (4)	C44D—C46D	1.630 (16)
C44C—C46C	1.568 (4)	C44D—C45D	1.652 (14)
C45C—H45D	0.9800	C45D—H45J	0.9800
C45C—H45E	0.9800	C45D—H45K	0.9800
C45C—H45F	0.9800	C45D—H45L	0.9800
C46C—H46D	0.9800	C46D—H46J	0.9800
C46C—H46E	0.9800	C46D—H46K	0.9800
C46C—H46F	0.9800	C46D—H46L	0.9800
C47C—H47D	0.9800	C47D—H47J	0.9800
C47C—H47E	0.9800	C47D—H47K	0.9800
C47C—H47F	0.9800	C47D—H47L	0.9800
C48A—H48A	0.9500	C48B—H48B	0.9500
C49A—C50A	1.373 (2)	C49B—C62B	1.371 (2)
C49A—C62A	1.377 (2)	C49B—C50B	1.382 (2)
C50A—C51A	1.3998 (19)	C50B—C51B	1.3944 (19)
C50A—H50A	0.9500	C50B—H50B	0.9500
C51A—C56A	1.392 (2)	C51B—C56B	1.394 (2)
C51A—C52A	1.533 (2)	C51B—C52B	1.532 (2)
C52A—C54A	1.527 (2)	C52B—C55B	1.527 (3)
C52A—C53A	1.527 (2)	C52B—C54B	1.534 (2)
C52A—C55A	1.532 (2)	C52B—C53B	1.536 (3)
C53A—H53A	0.9800	C53B—H53D	0.9800
C53A—H53B	0.9800	C53B—H53E	0.9800
C53A—H53C	0.9800	C53B—H53F	0.9800
C54A—H54A	0.9800	C54B—H54D	0.9800
C54A—H54B	0.9800	C54B—H54E	0.9800
C54A—H54C	0.9800	C54B—H54F	0.9800
C55A—H55A	0.9800	C55B—H55D	0.9800
C55A—H55B	0.9800	C55B—H55E	0.9800
C55A—H55C	0.9800	C55B—H55F	0.9800
C56A—C57A	1.398 (2)	C56B—C57B	1.393 (2)
C56A—H56A	0.9500	C56B—H56B	0.9500
C57A—C62A	1.392 (2)	C57B—C62B	1.400 (2)
C57A—C58A	1.530 (2)	C57B—C58B	1.537 (2)
C57A—C58C	1.530 (2)	C57B—C58D	1.537 (2)
C58A—C60A	1.296 (6)	C58B—C60B	1.512 (4)
C58A—C61A	1.526 (3)	C58B—C59B	1.525 (3)
C58A—C59A	1.800 (7)	C58B—C61B	1.535 (3)
C59A—H59A	0.9800	C59B—H59G	0.9800
C59A—H59B	0.9800	C59B—H59H	0.9800
C59A—H59C	0.9800	C59B—H59I	0.9800
C60A—H60A	0.9800	C60B—H60G	0.9800
C60A—H60B	0.9800	C60B—H60H	0.9800
C60A—H60C	0.9800	C60B—H60I	0.9800
C61A—H61A	0.9800	C61B—H61G	0.9800
C61A—H61B	0.9800	C61B—H61H	0.9800
C61A—H61C	0.9800	C61B—H61I	0.9800
C58C—C59C	1.394 (5)	C58D—C60D	1.49 (2)
C58C—C61C	1.526 (3)	C58D—C61D	1.564 (17)
C58C—C60C	1.777 (8)	C58D—C59D	1.581 (14)
C59C—H59D	0.9800	C59D—H59J	0.9800
C59C—H59E	0.9800	C59D—H59K	0.9800
C59C—H59F	0.9800	C59D—H59L	0.9800
C60C—H60D	0.9800	C61D—H61J	0.9800
C60C—H60E	0.9800	C61D—H61K	0.9800
C60C—H60F	0.9800	C61D—H61L	0.9800
C61C—H61D	0.9800	C60D—H60J	0.9800
C61C—H61E	0.9800	C60D—H60K	0.9800
C61C—H61F	0.9800	C60D—H60L	0.9800
C62A—H62A	0.9500	C62B—H62B	0.9500
			
C1A—O1A—C7A	120.31 (10)	C1B—O1B—C7B	118.50 (11)
C2A—O2A—C21A	120.95 (10)	C2B—O2B—C21B	118.41 (11)
C3A—O3A—C35A	123.29 (10)	C3B—O3B—C35B	118.98 (10)
C4A—O4A—C49A	119.85 (10)	C4B—O4B—C49B	119.83 (11)
C4A—N1A—C1A	115.54 (11)	C4B—N1B—C1B	116.10 (11)
C1A—N2A—C5A	113.77 (11)	C1B—N2B—C5B	113.53 (12)
C2A—N3A—C3A	116.29 (11)	C2B—N3B—C3B	116.54 (11)
C3A—N4A—C6A	113.73 (11)	C3B—N4B—C6B	113.21 (12)
N2A—C1A—O1A	120.74 (11)	N2B—C1B—N1B	128.86 (13)
N2A—C1A—N1A	129.40 (12)	N2B—C1B—O1B	114.88 (12)
O1A—C1A—N1A	109.85 (11)	N1B—C1B—O1B	116.25 (11)
N3A—C2A—O2A	121.62 (12)	N3B—C2B—O2B	120.91 (12)
N3A—C2A—C5A	122.72 (12)	N3B—C2B—C5B	122.32 (12)
O2A—C2A—C5A	115.61 (11)	O2B—C2B—C5B	116.72 (12)
N4A—C3A—O3A	112.88 (11)	N4B—C3B—O3B	119.87 (12)
N4A—C3A—N3A	128.46 (12)	N4B—C3B—N3B	128.97 (12)
O3A—C3A—N3A	118.66 (11)	O3B—C3B—N3B	111.15 (11)
N1A—C4A—O4A	122.25 (12)	N1B—C4B—O4B	122.07 (12)
N1A—C4A—C6A	122.81 (12)	N1B—C4B—C6B	122.56 (12)
O4A—C4A—C6A	114.93 (11)	O4B—C4B—C6B	115.32 (12)
N2A—C5A—C6A	124.06 (12)	N2B—C5B—C6B	124.00 (12)
N2A—C5A—C2A	122.08 (11)	N2B—C5B—C2B	122.38 (12)
C6A—C5A—C2A	113.81 (11)	C6B—C5B—C2B	113.52 (12)
N4A—C6A—C5A	124.96 (12)	N4B—C6B—C5B	124.97 (12)
N4A—C6A—C4A	120.74 (11)	N4B—C6B—C4B	120.74 (12)
C5A—C6A—C4A	114.27 (11)	C5B—C6B—C4B	114.14 (12)
C20A—C7A—C8A	122.50 (12)	C8B—C7B—C20B	122.77 (12)
C20A—C7A—O1A	121.87 (12)	C8B—C7B—O1B	118.06 (12)
C8A—C7A—O1A	115.31 (12)	C20B—C7B—O1B	118.96 (12)
C7A—C8A—C9A	119.44 (13)	C7B—C8B—C9B	119.15 (13)
C7A—C8A—H8AA	120.3	C7B—C8B—H8BA	120.4
C9A—C8A—H8AA	120.3	C9B—C8B—H8BA	120.4
C8A—C9A—C14A	118.18 (13)	C14B—C9B—C8B	117.84 (13)
C8A—C9A—C10A	119.82 (13)	C14B—C9B—C10B	122.30 (12)
C14A—C9A—C10A	121.94 (12)	C8B—C9B—C10B	119.84 (13)
C13A—C10A—C11A	109.26 (18)	C12B—C10B—C13B	108.13 (15)
C13A—C10A—C12A	109.51 (15)	C12B—C10B—C11B	108.31 (16)
C11A—C10A—C12A	107.54 (17)	C13B—C10B—C11B	107.37 (16)
C13A—C10A—C9A	107.97 (13)	C12B—C10B—C9B	113.05 (14)
C11A—C10A—C9A	110.76 (14)	C13B—C10B—C9B	111.03 (13)
C12A—C10A—C9A	111.77 (13)	C11B—C10B—C9B	108.76 (14)
C10A—C11A—H11A	109.5	C10B—C11B—H11D	109.5
C10A—C11A—H11B	109.5	C10B—C11B—H11E	109.5
H11A—C11A—H11B	109.5	H11D—C11B—H11E	109.5
C10A—C11A—H11C	109.5	C10B—C11B—H11F	109.5
H11A—C11A—H11C	109.5	H11D—C11B—H11F	109.5
H11B—C11A—H11C	109.5	H11E—C11B—H11F	109.5
C10A—C12A—H12A	109.5	C10B—C12B—H12D	109.5
C10A—C12A—H12B	109.5	C10B—C12B—H12E	109.5
H12A—C12A—H12B	109.5	H12D—C12B—H12E	109.5
C10A—C12A—H12C	109.5	C10B—C12B—H12F	109.5
H12A—C12A—H12C	109.5	H12D—C12B—H12F	109.5
H12B—C12A—H12C	109.5	H12E—C12B—H12F	109.5
C10A—C13A—H13A	109.5	C10B—C13B—H13D	109.5
C10A—C13A—H13B	109.5	C10B—C13B—H13E	109.5
H13A—C13A—H13B	109.5	H13D—C13B—H13E	109.5
C10A—C13A—H13C	109.5	C10B—C13B—H13F	109.5
H13A—C13A—H13C	109.5	H13D—C13B—H13F	109.5
H13B—C13A—H13C	109.5	H13E—C13B—H13F	109.5
C9A—C14A—C15A	122.55 (12)	C9B—C14B—C15B	122.66 (12)
C9A—C14A—H14A	118.7	C9B—C14B—H14B	118.7
C15A—C14A—H14A	118.7	C15B—C14B—H14B	118.7
C14A—C15A—C20A	118.24 (12)	C20B—C15B—C14B	118.42 (13)
C14A—C15A—C16A	121.87 (12)	C20B—C15B—C16B	121.27 (13)
C20A—C15A—C16A	119.82 (12)	C14B—C15B—C16B	120.27 (12)
C17A—C16A—C15A	108.30 (12)	C19B—C16B—C18B	108.25 (14)
C17A—C16A—C19A	110.29 (13)	C19B—C16B—C15B	112.07 (11)
C15A—C16A—C19A	110.49 (12)	C18B—C16B—C15B	110.16 (13)
C17A—C16A—C18A	108.37 (13)	C19B—C16B—C17B	108.58 (13)
C15A—C16A—C18A	112.08 (12)	C18B—C16B—C17B	109.33 (14)
C19A—C16A—C18A	107.30 (13)	C15B—C16B—C17B	108.41 (13)
C16A—C17A—H17A	109.5	C16B—C17B—H17D	109.5
C16A—C17A—H17B	109.5	C16B—C17B—H17E	109.5
H17A—C17A—H17B	109.5	H17D—C17B—H17E	109.5
C16A—C17A—H17C	109.5	C16B—C17B—H17F	109.5
H17A—C17A—H17C	109.5	H17D—C17B—H17F	109.5
H17B—C17A—H17C	109.5	H17E—C17B—H17F	109.5
C16A—C18A—H18A	109.5	C16B—C18B—H18D	109.5
C16A—C18A—H18B	109.5	C16B—C18B—H18E	109.5
H18A—C18A—H18B	109.5	H18D—C18B—H18E	109.5
C16A—C18A—H18C	109.5	C16B—C18B—H18F	109.5
H18A—C18A—H18C	109.5	H18D—C18B—H18F	109.5
H18B—C18A—H18C	109.5	H18E—C18B—H18F	109.5
C16A—C19A—H19A	109.5	C16B—C19B—H19D	109.5
C16A—C19A—H19B	109.5	C16B—C19B—H19E	109.5
H19A—C19A—H19B	109.5	H19D—C19B—H19E	109.5
C16A—C19A—H19C	109.5	C16B—C19B—H19F	109.5
H19A—C19A—H19C	109.5	H19D—C19B—H19F	109.5
H19B—C19A—H19C	109.5	H19E—C19B—H19F	109.5
C7A—C20A—C15A	119.04 (13)	C15B—C20B—C7B	119.14 (13)
C7A—C20A—H20A	120.5	C15B—C20B—H20B	120.4
C15A—C20A—H20A	120.5	C7B—C20B—H20B	120.4
C34A—C21A—C22A	123.18 (13)	C22B—C21B—C34B	123.08 (13)
C34A—C21A—O2A	121.38 (12)	C22B—C21B—O2B	116.61 (12)
C22A—C21A—O2A	115.17 (12)	C34B—C21B—O2B	120.09 (13)
C21A—C22A—C23A	119.02 (14)	C21B—C22B—C23B	119.50 (13)
C21A—C22A—H22A	120.5	C21B—C22B—H22B	120.3
C23A—C22A—H22A	120.5	C23B—C22B—H22B	120.3
C22A—C23A—C28A	118.01 (13)	C28B—C23B—C22B	117.57 (14)
C22A—C23A—C24A	121.50 (14)	C28B—C23B—C24B	122.04 (13)
C28A—C23A—C24A	120.49 (13)	C22B—C23B—C24B	120.40 (14)
C27A—C24A—C26A	108.70 (18)	C28B—C23B—C24D	122.04 (13)
C27A—C24A—C25A	109.59 (17)	C22B—C23B—C24D	120.40 (14)
C26A—C24A—C25A	107.05 (18)	C25B—C24B—C23B	110.66 (17)
C27A—C24A—C23A	109.76 (15)	C25B—C24B—C26B	111.5 (2)
C26A—C24A—C23A	112.41 (14)	C23B—C24B—C26B	109.41 (14)
C25A—C24A—C23A	109.26 (14)	C25B—C24B—C27B	108.04 (19)
C24A—C25A—H25A	109.5	C23B—C24B—C27B	112.46 (14)
C24A—C25A—H25B	109.5	C26B—C24B—C27B	104.64 (18)
H25A—C25A—H25B	109.5	C24B—C25B—H25D	109.5
C24A—C25A—H25C	109.5	C24B—C25B—H25E	109.5
H25A—C25A—H25C	109.5	H25D—C25B—H25E	109.5
H25B—C25A—H25C	109.5	C24B—C25B—H25F	109.5
C24A—C26A—H26A	109.5	H25D—C25B—H25F	109.5
C24A—C26A—H26B	109.5	H25E—C25B—H25F	109.5
H26A—C26A—H26B	109.5	C24B—C26B—H26D	109.5
C24A—C26A—H26C	109.5	C24B—C26B—H26E	109.5
H26A—C26A—H26C	109.5	H26D—C26B—H26E	109.5
H26B—C26A—H26C	109.5	C24B—C26B—H26F	109.5
C24A—C27A—H27A	109.5	H26D—C26B—H26F	109.5
C24A—C27A—H27B	109.5	H26E—C26B—H26F	109.5
H27A—C27A—H27B	109.5	C24B—C27B—H27D	109.5
C24A—C27A—H27C	109.5	C24B—C27B—H27E	109.5
H27A—C27A—H27C	109.5	H27D—C27B—H27E	109.5
H27B—C27A—H27C	109.5	C24B—C27B—H27F	109.5
C29A—C28A—C23A	122.67 (13)	H27D—C27B—H27F	109.5
C29A—C28A—H28A	118.7	H27E—C27B—H27F	109.5
C23A—C28A—H28A	118.7	C26D—C24D—C27D	119.4 (11)
C28A—C29A—C34A	118.37 (14)	C26D—C24D—C23B	108.6 (9)
C28A—C29A—C30A	123.32 (13)	C27D—C24D—C23B	107.7 (5)
C34A—C29A—C30A	118.27 (14)	C26D—C24D—C25D	115.7 (11)
C31A—C30A—C29A	112.41 (15)	C27D—C24D—C25D	98.8 (6)
C31A—C30A—C32A	108.39 (14)	C23B—C24D—C25D	105.4 (4)
C29A—C30A—C32A	108.28 (13)	C24D—C25D—H25G	109.5
C31A—C30A—C33A	108.32 (14)	C24D—C25D—H25H	109.5
C29A—C30A—C33A	110.42 (12)	H25G—C25D—H25H	109.5
C32A—C30A—C33A	108.95 (16)	C24D—C25D—H25I	109.5
C30A—C31A—H31A	109.5	H25G—C25D—H25I	109.5
C30A—C31A—H31B	109.5	H25H—C25D—H25I	109.5
H31A—C31A—H31B	109.5	C24D—C26D—H26G	109.5
C30A—C31A—H31C	109.5	C24D—C26D—H26H	109.5
H31A—C31A—H31C	109.5	H26G—C26D—H26H	109.5
H31B—C31A—H31C	109.5	C24D—C26D—H26I	109.5
C30A—C32A—H32A	109.5	H26G—C26D—H26I	109.5
C30A—C32A—H32B	109.5	H26H—C26D—H26I	109.5
H32A—C32A—H32B	109.5	C24D—C27D—H27G	109.5
C30A—C32A—H32C	109.5	C24D—C27D—H27H	109.5
H32A—C32A—H32C	109.5	H27G—C27D—H27H	109.5
H32B—C32A—H32C	109.5	C24D—C27D—H27I	109.5
C30A—C33A—H33A	109.5	H27G—C27D—H27I	109.5
C30A—C33A—H33B	109.5	H27H—C27D—H27I	109.5
H33A—C33A—H33B	109.5	C23B—C28B—C29B	122.68 (13)
C30A—C33A—H33C	109.5	C23B—C28B—H28B	118.7
H33A—C33A—H33C	109.5	C29B—C28B—H28B	118.7
H33B—C33A—H33C	109.5	C34B—C29B—C28B	118.66 (13)
C21A—C34A—C29A	118.72 (13)	C34B—C29B—C30B	121.12 (14)
C21A—C34A—H34A	120.6	C28B—C29B—C30B	120.13 (13)
C29A—C34A—H34A	120.6	C31B—C30B—C33B	108.54 (14)
C48A—C35A—C36A	122.39 (13)	C31B—C30B—C29B	111.62 (13)
C48A—C35A—O3A	114.45 (12)	C33B—C30B—C29B	110.96 (13)
C36A—C35A—O3A	122.80 (12)	C31B—C30B—C32B	108.89 (16)
C35A—C36A—C37A	119.04 (14)	C33B—C30B—C32B	109.70 (14)
C35A—C36A—H36A	120.5	C29B—C30B—C32B	107.08 (13)
C37A—C36A—H36A	120.5	C30B—C31B—H31D	109.5
C36A—C37A—C42A	118.43 (14)	C30B—C31B—H31E	109.5
C36A—C37A—C38A	120.77 (15)	H31D—C31B—H31E	109.5
C42A—C37A—C38A	120.80 (14)	C30B—C31B—H31F	109.5
C36A—C37A—C38C	120.77 (15)	H31D—C31B—H31F	109.5
C42A—C37A—C38C	120.80 (14)	H31E—C31B—H31F	109.5
C41A—C38A—C37A	113.13 (19)	C30B—C32B—H32D	109.5
C41A—C38A—C39A	111.8 (2)	C30B—C32B—H32E	109.5
C37A—C38A—C39A	112.68 (17)	H32D—C32B—H32E	109.5
C41A—C38A—C40A	109.7 (2)	C30B—C32B—H32F	109.5
C37A—C38A—C40A	107.07 (17)	H32D—C32B—H32F	109.5
C39A—C38A—C40A	101.7 (2)	H32E—C32B—H32F	109.5
C38A—C39A—H39A	109.5	C30B—C33B—H33D	109.5
C38A—C39A—H39B	109.5	C30B—C33B—H33E	109.5
H39A—C39A—H39B	109.5	H33D—C33B—H33E	109.5
C38A—C39A—H39C	109.5	C30B—C33B—H33F	109.5
H39A—C39A—H39C	109.5	H33D—C33B—H33F	109.5
H39B—C39A—H39C	109.5	H33E—C33B—H33F	109.5
C38A—C40A—H40A	109.5	C21B—C34B—C29B	118.50 (14)
C38A—C40A—H40B	109.5	C21B—C34B—H34B	120.7
H40A—C40A—H40B	109.5	C29B—C34B—H34B	120.7
C38A—C40A—H40C	109.5	C36B—C35B—C48B	122.63 (13)
H40A—C40A—H40C	109.5	C36B—C35B—O3B	116.67 (13)
H40B—C40A—H40C	109.5	C48B—C35B—O3B	120.40 (13)
C38A—C41A—H41A	109.5	C35B—C36B—C37B	119.28 (14)
C38A—C41A—H41B	109.5	C35B—C36B—H36B	120.4
H41A—C41A—H41B	109.5	C37B—C36B—H36B	120.4
C38A—C41A—H41C	109.5	C42B—C37B—C36B	118.43 (14)
H41A—C41A—H41C	109.5	C42B—C37B—C38B	123.37 (14)
H41B—C41A—H41C	109.5	C36B—C37B—C38B	118.20 (14)
C40C—C38C—C37A	112.5 (2)	C41B—C38B—C37B	109.20 (14)
C40C—C38C—C41C	115.4 (3)	C41B—C38B—C40B	109.17 (17)
C37A—C38C—C41C	111.0 (2)	C37B—C38B—C40B	109.25 (15)
C40C—C38C—C39C	112.1 (3)	C41B—C38B—C39B	109.85 (17)
C37A—C38C—C39C	104.71 (18)	C37B—C38B—C39B	111.89 (15)
C41C—C38C—C39C	99.9 (3)	C40B—C38B—C39B	107.43 (15)
C38C—C39C—H39D	109.5	C38B—C39B—H39G	109.5
C38C—C39C—H39E	109.5	C38B—C39B—H39H	109.5
H39D—C39C—H39E	109.5	H39G—C39B—H39H	109.5
C38C—C39C—H39F	109.5	C38B—C39B—H39I	109.5
H39D—C39C—H39F	109.5	H39G—C39B—H39I	109.5
H39E—C39C—H39F	109.5	H39H—C39B—H39I	109.5
C38C—C40C—H40D	109.5	C38B—C40B—H40G	109.5
C38C—C40C—H40E	109.5	C38B—C40B—H40H	109.5
H40D—C40C—H40E	109.5	H40G—C40B—H40H	109.5
C38C—C40C—H40F	109.5	C38B—C40B—H40I	109.5
H40D—C40C—H40F	109.5	H40G—C40B—H40I	109.5
H40E—C40C—H40F	109.5	H40H—C40B—H40I	109.5
C38C—C41C—H41D	109.5	C38B—C41B—H41G	109.5
C38C—C41C—H41E	109.5	C38B—C41B—H41H	109.5
H41D—C41C—H41E	109.5	H41G—C41B—H41H	109.5
C38C—C41C—H41F	109.5	C38B—C41B—H41I	109.5
H41D—C41C—H41F	109.5	H41G—C41B—H41I	109.5
H41E—C41C—H41F	109.5	H41H—C41B—H41I	109.5
C43A—C42A—C37A	122.50 (14)	C37B—C42B—C43B	122.38 (14)
C43A—C42A—H42A	118.8	C37B—C42B—H42B	118.8
C37A—C42A—H42A	118.8	C43B—C42B—H42B	118.8
C48A—C43A—C42A	118.07 (14)	C42B—C43B—C48B	118.28 (14)
C48A—C43A—C44A	120.41 (14)	C42B—C43B—C44B	121.80 (14)
C42A—C43A—C44A	121.52 (14)	C48B—C43B—C44B	119.91 (15)
C48A—C43A—C44C	120.41 (14)	C42B—C43B—C44D	121.80 (14)
C42A—C43A—C44C	121.52 (14)	C48B—C43B—C44D	119.91 (15)
C45A—C44A—C43A	110.6 (2)	C46B—C44B—C47B	110.0 (2)
C45A—C44A—C46A	108.5 (3)	C46B—C44B—C43B	109.38 (15)
C43A—C44A—C46A	113.82 (17)	C47B—C44B—C43B	108.78 (15)
C45A—C44A—C47A	110.1 (3)	C46B—C44B—C45B	109.6 (2)
C43A—C44A—C47A	110.60 (17)	C47B—C44B—C45B	107.8 (2)
C46A—C44A—C47A	103.0 (2)	C43B—C44B—C45B	111.23 (16)
C44A—C45A—H45A	109.5	C44B—C45B—H45G	109.5
C44A—C45A—H45B	109.5	C44B—C45B—H45H	109.5
H45A—C45A—H45B	109.5	H45G—C45B—H45H	109.5
C44A—C45A—H45C	109.5	C44B—C45B—H45I	109.5
H45A—C45A—H45C	109.5	H45G—C45B—H45I	109.5
H45B—C45A—H45C	109.5	H45H—C45B—H45I	109.5
C44A—C46A—H46A	109.5	C44B—C46B—H46G	109.5
C44A—C46A—H46B	109.5	C44B—C46B—H46H	109.5
H46A—C46A—H46B	109.5	H46G—C46B—H46H	109.5
C44A—C46A—H46C	109.5	C44B—C46B—H46I	109.5
H46A—C46A—H46C	109.5	H46G—C46B—H46I	109.5
H46B—C46A—H46C	109.5	H46H—C46B—H46I	109.5
C44A—C47A—H47A	109.5	C44B—C47B—H47G	109.5
C44A—C47A—H47B	109.5	C44B—C47B—H47H	109.5
H47A—C47A—H47B	109.5	H47G—C47B—H47H	109.5
C44A—C47A—H47C	109.5	C44B—C47B—H47I	109.5
H47A—C47A—H47C	109.5	H47G—C47B—H47I	109.5
H47B—C47A—H47C	109.5	H47H—C47B—H47I	109.5
C47C—C44C—C43A	108.55 (18)	C47D—C44D—C43B	116.4 (6)
C47C—C44C—C45C	113.0 (3)	C47D—C44D—C46D	105.5 (11)
C43A—C44C—C45C	109.4 (2)	C43B—C44D—C46D	116.4 (6)
C47C—C44C—C46C	112.1 (3)	C47D—C44D—C45D	106.3 (10)
C43A—C44C—C46C	106.56 (17)	C43B—C44D—C45D	110.8 (5)
C45C—C44C—C46C	107.0 (2)	C46D—C44D—C45D	99.7 (10)
C44C—C45C—H45D	109.5	C44D—C45D—H45J	109.5
C44C—C45C—H45E	109.5	C44D—C45D—H45K	109.5
H45D—C45C—H45E	109.5	H45J—C45D—H45K	109.5
C44C—C45C—H45F	109.5	C44D—C45D—H45L	109.5
H45D—C45C—H45F	109.5	H45J—C45D—H45L	109.5
H45E—C45C—H45F	109.5	H45K—C45D—H45L	109.5
C44C—C46C—H46D	109.5	C44D—C46D—H46J	109.5
C44C—C46C—H46E	109.5	C44D—C46D—H46K	109.5
H46D—C46C—H46E	109.5	H46J—C46D—H46K	109.5
C44C—C46C—H46F	109.5	C44D—C46D—H46L	109.5
H46D—C46C—H46F	109.5	H46J—C46D—H46L	109.5
H46E—C46C—H46F	109.5	H46K—C46D—H46L	109.5
C44C—C47C—H47D	109.5	C44D—C47D—H47J	109.5
C44C—C47C—H47E	109.5	C44D—C47D—H47K	109.5
H47D—C47C—H47E	109.5	H47J—C47D—H47K	109.5
C44C—C47C—H47F	109.5	C44D—C47D—H47L	109.5
H47D—C47C—H47F	109.5	H47J—C47D—H47L	109.5
H47E—C47C—H47F	109.5	H47K—C47D—H47L	109.5
C35A—C48A—C43A	119.56 (13)	C35B—C48B—C43B	118.96 (14)
C35A—C48A—H48A	120.2	C35B—C48B—H48B	120.5
C43A—C48A—H48A	120.2	C43B—C48B—H48B	120.5
C50A—C49A—C62A	123.65 (13)	C62B—C49B—C50B	123.30 (13)
C50A—C49A—O4A	117.73 (13)	C62B—C49B—O4B	115.97 (13)
C62A—C49A—O4A	118.12 (13)	C50B—C49B—O4B	120.45 (13)
C49A—C50A—C51A	118.82 (13)	C49B—C50B—C51B	118.33 (14)
C49A—C50A—H50A	120.6	C49B—C50B—H50B	120.8
C51A—C50A—H50A	120.6	C51B—C50B—H50B	120.8
C56A—C51A—C50A	117.69 (13)	C56B—C51B—C50B	118.48 (15)
C56A—C51A—C52A	121.57 (13)	C56B—C51B—C52B	119.85 (13)
C50A—C51A—C52A	120.58 (13)	C50B—C51B—C52B	121.65 (15)
C54A—C52A—C53A	108.21 (16)	C55B—C52B—C51B	111.92 (13)
C54A—C52A—C55A	108.43 (15)	C55B—C52B—C54B	108.22 (18)
C53A—C52A—C55A	109.05 (15)	C51B—C52B—C54B	109.72 (14)
C54A—C52A—C51A	111.70 (13)	C55B—C52B—C53B	108.33 (17)
C53A—C52A—C51A	111.42 (13)	C51B—C52B—C53B	108.88 (16)
C55A—C52A—C51A	107.96 (14)	C54B—C52B—C53B	109.75 (16)
C52A—C53A—H53A	109.5	C52B—C53B—H53D	109.5
C52A—C53A—H53B	109.5	C52B—C53B—H53E	109.5
H53A—C53A—H53B	109.5	H53D—C53B—H53E	109.5
C52A—C53A—H53C	109.5	C52B—C53B—H53F	109.5
H53A—C53A—H53C	109.5	H53D—C53B—H53F	109.5
H53B—C53A—H53C	109.5	H53E—C53B—H53F	109.5
C52A—C54A—H54A	109.5	C52B—C54B—H54D	109.5
C52A—C54A—H54B	109.5	C52B—C54B—H54E	109.5
H54A—C54A—H54B	109.5	H54D—C54B—H54E	109.5
C52A—C54A—H54C	109.5	C52B—C54B—H54F	109.5
H54A—C54A—H54C	109.5	H54D—C54B—H54F	109.5
H54B—C54A—H54C	109.5	H54E—C54B—H54F	109.5
C52A—C55A—H55A	109.5	C52B—C55B—H55D	109.5
C52A—C55A—H55B	109.5	C52B—C55B—H55E	109.5
H55A—C55A—H55B	109.5	H55D—C55B—H55E	109.5
C52A—C55A—H55C	109.5	C52B—C55B—H55F	109.5
H55A—C55A—H55C	109.5	H55D—C55B—H55F	109.5
H55B—C55A—H55C	109.5	H55E—C55B—H55F	109.5
C51A—C56A—C57A	123.15 (14)	C57B—C56B—C51B	123.06 (13)
C51A—C56A—H56A	118.4	C57B—C56B—H56B	118.5
C57A—C56A—H56A	118.4	C51B—C56B—H56B	118.5
C62A—C57A—C56A	117.95 (15)	C56B—C57B—C62B	117.36 (15)
C62A—C57A—C58A	121.68 (15)	C56B—C57B—C58B	122.89 (14)
C56A—C57A—C58A	120.37 (15)	C62B—C57B—C58B	119.73 (15)
C62A—C57A—C58C	121.68 (15)	C56B—C57B—C58D	122.89 (14)
C56A—C57A—C58C	120.37 (15)	C62B—C57B—C58D	119.73 (15)
C60A—C58A—C61A	113.2 (4)	C60B—C58B—C59B	110.0 (3)
C60A—C58A—C57A	119.5 (3)	C60B—C58B—C61B	108.8 (2)
C61A—C58A—C57A	112.71 (16)	C59B—C58B—C61B	106.9 (2)
C60A—C58A—C59A	107.2 (4)	C60B—C58B—C57B	109.18 (17)
C61A—C58A—C59A	98.4 (3)	C59B—C58B—C57B	112.17 (19)
C57A—C58A—C59A	102.6 (3)	C61B—C58B—C57B	109.72 (16)
C58A—C59A—H59A	109.5	C58B—C59B—H59G	109.5
C58A—C59A—H59B	109.5	C58B—C59B—H59H	109.5
H59A—C59A—H59B	109.5	H59G—C59B—H59H	109.5
C58A—C59A—H59C	109.5	C58B—C59B—H59I	109.5
H59A—C59A—H59C	109.5	H59G—C59B—H59I	109.5
H59B—C59A—H59C	109.5	H59H—C59B—H59I	109.5
C58A—C60A—H60A	109.5	C58B—C60B—H60G	109.5
C58A—C60A—H60B	109.5	C58B—C60B—H60H	109.5
H60A—C60A—H60B	109.5	H60G—C60B—H60H	109.5
C58A—C60A—H60C	109.5	C58B—C60B—H60I	109.5
H60A—C60A—H60C	109.5	H60G—C60B—H60I	109.5
H60B—C60A—H60C	109.5	H60H—C60B—H60I	109.5
C58A—C61A—H61A	109.5	C58B—C61B—H61G	109.5
C58A—C61A—H61B	109.5	C58B—C61B—H61H	109.5
H61A—C61A—H61B	109.5	H61G—C61B—H61H	109.5
C58A—C61A—H61C	109.5	C58B—C61B—H61I	109.5
H61A—C61A—H61C	109.5	H61G—C61B—H61I	109.5
H61B—C61A—H61C	109.5	H61H—C61B—H61I	109.5
C59C—C58C—C61C	116.1 (4)	C60D—C58D—C57B	114.3 (8)
C59C—C58C—C57A	114.9 (3)	C60D—C58D—C61D	108.8 (11)
C61C—C58C—C57A	112.71 (16)	C57B—C58D—C61D	109.2 (7)
C59C—C58C—C60C	104.4 (4)	C60D—C58D—C59D	110.9 (10)
C61C—C58C—C60C	105.1 (2)	C57B—C58D—C59D	107.8 (6)
C57A—C58C—C60C	101.4 (3)	C61D—C58D—C59D	105.5 (9)
C58C—C59C—H59D	109.5	C58D—C59D—H59J	109.5
C58C—C59C—H59E	109.5	C58D—C59D—H59K	109.5
H59D—C59C—H59E	109.5	H59J—C59D—H59K	109.5
C58C—C59C—H59F	109.5	C58D—C59D—H59L	109.5
H59D—C59C—H59F	109.5	H59J—C59D—H59L	109.5
H59E—C59C—H59F	109.5	H59K—C59D—H59L	109.5
C58C—C60C—H60D	109.5	C58D—C61D—H61J	109.5
C58C—C60C—H60E	109.5	C58D—C61D—H61K	109.5
H60D—C60C—H60E	109.5	H61J—C61D—H61K	109.5
C58C—C60C—H60F	109.5	C58D—C61D—H61L	109.5
H60D—C60C—H60F	109.5	H61J—C61D—H61L	109.5
H60E—C60C—H60F	109.5	H61K—C61D—H61L	109.5
C58C—C61C—H61D	109.5	C58D—C60D—H60J	109.5
C58C—C61C—H61E	109.5	C58D—C60D—H60K	109.5
H61D—C61C—H61E	109.5	H60J—C60D—H60K	109.5
C58C—C61C—H61F	109.5	C58D—C60D—H60L	109.5
H61D—C61C—H61F	109.5	H60J—C60D—H60L	109.5
H61E—C61C—H61F	109.5	H60K—C60D—H60L	109.5
C49A—C62A—C57A	118.73 (14)	C49B—C62B—C57B	119.46 (15)
C49A—C62A—H62A	120.6	C49B—C62B—H62B	120.3
C57A—C62A—H62A	120.6	C57B—C62B—H62B	120.3
			
C5A—N2A—C1A—O1A	−177.74 (12)	C5B—N2B—C1B—N1B	−6.5 (2)
C5A—N2A—C1A—N1A	3.1 (2)	C5B—N2B—C1B—O1B	173.42 (11)
C7A—O1A—C1A—N2A	−0.21 (19)	C4B—N1B—C1B—N2B	6.0 (2)
C7A—O1A—C1A—N1A	179.13 (11)	C4B—N1B—C1B—O1B	−173.88 (12)
C4A—N1A—C1A—N2A	−0.8 (2)	C7B—O1B—C1B—N2B	156.53 (12)
C4A—N1A—C1A—O1A	179.98 (11)	C7B—O1B—C1B—N1B	−23.52 (18)
C3A—N3A—C2A—O2A	−177.13 (12)	C3B—N3B—C2B—O2B	−176.22 (12)
C3A—N3A—C2A—C5A	0.12 (19)	C3B—N3B—C2B—C5B	1.12 (19)
C21A—O2A—C2A—N3A	−4.47 (19)	C21B—O2B—C2B—N3B	9.08 (19)
C21A—O2A—C2A—C5A	178.09 (12)	C21B—O2B—C2B—C5B	−168.40 (12)
C6A—N4A—C3A—O3A	−177.99 (11)	C6B—N4B—C3B—O3B	177.58 (12)
C6A—N4A—C3A—N3A	1.72 (19)	C6B—N4B—C3B—N3B	−3.9 (2)
C35A—O3A—C3A—N4A	176.29 (12)	C35B—O3B—C3B—N4B	9.33 (19)
C35A—O3A—C3A—N3A	−3.46 (19)	C35B—O3B—C3B—N3B	−169.46 (12)
C2A—N3A—C3A—N4A	−1.7 (2)	C2B—N3B—C3B—N4B	4.6 (2)
C2A—N3A—C3A—O3A	178.04 (12)	C2B—N3B—C3B—O3B	−176.77 (12)
C1A—N1A—C4A—O4A	177.01 (12)	C1B—N1B—C4B—O4B	−174.77 (12)
C1A—N1A—C4A—C6A	−2.93 (19)	C1B—N1B—C4B—C6B	2.30 (19)
C49A—O4A—C4A—N1A	8.39 (19)	C49B—O4B—C4B—N1B	11.0 (2)
C49A—O4A—C4A—C6A	−171.67 (11)	C49B—O4B—C4B—C6B	−166.32 (12)
C1A—N2A—C5A—C6A	−1.81 (19)	C1B—N2B—C5B—C6B	−1.23 (19)
C1A—N2A—C5A—C2A	175.64 (12)	C1B—N2B—C5B—C2B	174.94 (12)
N3A—C2A—C5A—N2A	−176.69 (12)	N3B—C2B—C5B—N2B	177.23 (13)
O2A—C2A—C5A—N2A	0.71 (19)	O2B—C2B—C5B—N2B	−5.34 (19)
N3A—C2A—C5A—C6A	1.00 (19)	N3B—C2B—C5B—C6B	−6.24 (19)
O2A—C2A—C5A—C6A	178.40 (11)	O2B—C2B—C5B—C6B	171.20 (12)
C3A—N4A—C6A—C5A	−0.31 (19)	C3B—N4B—C6B—C5B	−2.54 (19)
C3A—N4A—C6A—C4A	177.55 (12)	C3B—N4B—C6B—C4B	172.78 (12)
N2A—C5A—C6A—N4A	176.73 (12)	N2B—C5B—C6B—N4B	−176.43 (12)
C2A—C5A—C6A—N4A	−0.91 (19)	C2B—C5B—C6B—N4B	7.10 (19)
N2A—C5A—C6A—C4A	−1.25 (19)	N2B—C5B—C6B—C4B	7.97 (19)
C2A—C5A—C6A—C4A	−178.89 (11)	C2B—C5B—C6B—C4B	−168.50 (11)
N1A—C4A—C6A—N4A	−174.28 (12)	N1B—C4B—C6B—N4B	175.70 (12)
O4A—C4A—C6A—N4A	5.77 (18)	O4B—C4B—C6B—N4B	−7.04 (18)
N1A—C4A—C6A—C5A	3.79 (19)	N1B—C4B—C6B—C5B	−8.50 (19)
O4A—C4A—C6A—C5A	−176.15 (11)	O4B—C4B—C6B—C5B	168.76 (11)
C1A—O1A—C7A—C20A	63.05 (18)	C1B—O1B—C7B—C8B	129.85 (14)
C1A—O1A—C7A—C8A	−123.29 (13)	C1B—O1B—C7B—C20B	−55.20 (18)
C20A—C7A—C8A—C9A	1.3 (2)	C20B—C7B—C8B—C9B	−1.0 (2)
O1A—C7A—C8A—C9A	−172.29 (12)	O1B—C7B—C8B—C9B	173.78 (13)
C7A—C8A—C9A—C14A	−2.2 (2)	C7B—C8B—C9B—C14B	1.2 (2)
C7A—C8A—C9A—C10A	−179.46 (13)	C7B—C8B—C9B—C10B	−177.02 (14)
C8A—C9A—C10A—C13A	77.78 (19)	C14B—C9B—C10B—C12B	6.8 (2)
C14A—C9A—C10A—C13A	−99.40 (18)	C8B—C9B—C10B—C12B	−175.00 (17)
C8A—C9A—C10A—C11A	−41.8 (2)	C14B—C9B—C10B—C13B	128.55 (17)
C14A—C9A—C10A—C11A	141.00 (18)	C8B—C9B—C10B—C13B	−53.3 (2)
C8A—C9A—C10A—C12A	−161.72 (16)	C14B—C9B—C10B—C11B	−113.52 (17)
C14A—C9A—C10A—C12A	21.1 (2)	C8B—C9B—C10B—C11B	64.66 (19)
C8A—C9A—C14A—C15A	1.1 (2)	C8B—C9B—C14B—C15B	−0.1 (2)
C10A—C9A—C14A—C15A	178.34 (13)	C10B—C9B—C14B—C15B	178.10 (14)
C9A—C14A—C15A—C20A	0.8 (2)	C9B—C14B—C15B—C20B	−1.3 (2)
C9A—C14A—C15A—C16A	177.64 (13)	C9B—C14B—C15B—C16B	−179.09 (14)
C14A—C15A—C16A—C17A	−110.71 (16)	C20B—C15B—C16B—C19B	12.6 (2)
C20A—C15A—C16A—C17A	66.03 (17)	C14B—C15B—C16B—C19B	−169.68 (14)
C14A—C15A—C16A—C19A	128.39 (15)	C20B—C15B—C16B—C18B	133.19 (16)
C20A—C15A—C16A—C19A	−54.87 (18)	C14B—C15B—C16B—C18B	−49.1 (2)
C14A—C15A—C16A—C18A	8.8 (2)	C20B—C15B—C16B—C17B	−107.21 (16)
C20A—C15A—C16A—C18A	−174.47 (13)	C14B—C15B—C16B—C17B	70.50 (18)
C8A—C7A—C20A—C15A	0.7 (2)	C14B—C15B—C20B—C7B	1.6 (2)
O1A—C7A—C20A—C15A	173.87 (12)	C16B—C15B—C20B—C7B	179.34 (13)
C14A—C15A—C20A—C7A	−1.7 (2)	C8B—C7B—C20B—C15B	−0.5 (2)
C16A—C15A—C20A—C7A	−178.58 (12)	O1B—C7B—C20B—C15B	−175.19 (13)
C2A—O2A—C21A—C34A	−52.95 (18)	C2B—O2B—C21B—C22B	113.55 (14)
C2A—O2A—C21A—C22A	132.85 (13)	C2B—O2B—C21B—C34B	−71.65 (17)
C34A—C21A—C22A—C23A	1.1 (2)	C34B—C21B—C22B—C23B	0.7 (2)
O2A—C21A—C22A—C23A	175.16 (12)	O2B—C21B—C22B—C23B	175.38 (13)
C21A—C22A—C23A—C28A	0.3 (2)	C21B—C22B—C23B—C28B	−1.3 (2)
C21A—C22A—C23A—C24A	179.98 (13)	C21B—C22B—C23B—C24B	178.93 (14)
C22A—C23A—C24A—C27A	123.92 (19)	C21B—C22B—C23B—C24D	178.93 (14)
C28A—C23A—C24A—C27A	−56.4 (2)	C28B—C23B—C24B—C25B	−117.1 (2)
C22A—C23A—C24A—C26A	2.8 (2)	C22B—C23B—C24B—C25B	62.6 (2)
C28A—C23A—C24A—C26A	−177.56 (16)	C28B—C23B—C24B—C26B	119.63 (19)
C22A—C23A—C24A—C25A	−115.88 (18)	C22B—C23B—C24B—C26B	−60.6 (2)
C28A—C23A—C24A—C25A	63.8 (2)	C28B—C23B—C24B—C27B	3.8 (2)
C22A—C23A—C28A—C29A	−1.2 (2)	C22B—C23B—C24B—C27B	−176.42 (17)
C24A—C23A—C28A—C29A	179.14 (14)	C28B—C23B—C24D—C26D	82.4 (10)
C23A—C28A—C29A—C34A	0.7 (2)	C22B—C23B—C24D—C26D	−97.8 (10)
C23A—C28A—C29A—C30A	−176.86 (14)	C28B—C23B—C24D—C27D	−48.2 (6)
C28A—C29A—C30A—C31A	−14.1 (2)	C22B—C23B—C24D—C27D	131.6 (5)
C34A—C29A—C30A—C31A	168.33 (14)	C28B—C23B—C24D—C25D	−152.9 (5)
C28A—C29A—C30A—C32A	105.59 (18)	C22B—C23B—C24D—C25D	26.8 (5)
C34A—C29A—C30A—C32A	−71.96 (18)	C22B—C23B—C28B—C29B	0.7 (2)
C28A—C29A—C30A—C33A	−135.21 (16)	C24B—C23B—C28B—C29B	−179.56 (14)
C34A—C29A—C30A—C33A	47.24 (19)	C24D—C23B—C28B—C29B	−179.56 (14)
C22A—C21A—C34A—C29A	−1.6 (2)	C23B—C28B—C29B—C34B	0.5 (2)
O2A—C21A—C34A—C29A	−175.33 (12)	C23B—C28B—C29B—C30B	−176.02 (14)
C28A—C29A—C34A—C21A	0.7 (2)	C34B—C29B—C30B—C31B	21.7 (2)
C30A—C29A—C34A—C21A	178.37 (13)	C28B—C29B—C30B—C31B	−161.82 (15)
C3A—O3A—C35A—C48A	133.98 (13)	C34B—C29B—C30B—C33B	142.92 (15)
C3A—O3A—C35A—C36A	−52.72 (19)	C28B—C29B—C30B—C33B	−40.6 (2)
C48A—C35A—C36A—C37A	−0.5 (2)	C34B—C29B—C30B—C32B	−97.38 (17)
O3A—C35A—C36A—C37A	−173.29 (13)	C28B—C29B—C30B—C32B	79.08 (18)
C35A—C36A—C37A—C42A	1.1 (2)	C22B—C21B—C34B—C29B	0.5 (2)
C35A—C36A—C37A—C38A	−178.76 (15)	O2B—C21B—C34B—C29B	−173.98 (12)
C35A—C36A—C37A—C38C	−178.76 (15)	C28B—C29B—C34B—C21B	−1.1 (2)
C36A—C37A—C38A—C41A	−122.0 (2)	C30B—C29B—C34B—C21B	175.43 (13)
C42A—C37A—C38A—C41A	58.1 (3)	C3B—O3B—C35B—C36B	−115.06 (14)
C36A—C37A—C38A—C39A	6.0 (3)	C3B—O3B—C35B—C48B	71.06 (17)
C42A—C37A—C38A—C39A	−173.9 (2)	C48B—C35B—C36B—C37B	1.6 (2)
C36A—C37A—C38A—C40A	117.0 (2)	O3B—C35B—C36B—C37B	−172.17 (13)
C42A—C37A—C38A—C40A	−62.9 (2)	C35B—C36B—C37B—C42B	−1.6 (2)
C36A—C37A—C38C—C40C	62.6 (3)	C35B—C36B—C37B—C38B	178.51 (14)
C42A—C37A—C38C—C40C	−117.3 (3)	C42B—C37B—C38B—C41B	−116.71 (18)
C36A—C37A—C38C—C41C	−166.3 (3)	C36B—C37B—C38B—C41B	63.22 (19)
C42A—C37A—C38C—C41C	13.8 (3)	C42B—C37B—C38B—C40B	123.96 (18)
C36A—C37A—C38C—C39C	−59.4 (3)	C36B—C37B—C38B—C40B	−56.1 (2)
C42A—C37A—C38C—C39C	120.7 (2)	C42B—C37B—C38B—C39B	5.1 (2)
C36A—C37A—C42A—C43A	−0.7 (2)	C36B—C37B—C38B—C39B	−174.95 (17)
C38A—C37A—C42A—C43A	179.23 (15)	C36B—C37B—C42B—C43B	0.0 (2)
C38C—C37A—C42A—C43A	179.23 (15)	C38B—C37B—C42B—C43B	179.93 (16)
C37A—C42A—C43A—C48A	−0.4 (2)	C37B—C42B—C43B—C48B	1.6 (2)
C37A—C42A—C43A—C44A	179.48 (15)	C37B—C42B—C43B—C44B	−179.68 (15)
C37A—C42A—C43A—C44C	179.48 (15)	C37B—C42B—C43B—C44D	−179.68 (15)
C48A—C43A—C44A—C45A	−124.3 (3)	C42B—C43B—C44B—C46B	−115.3 (2)
C42A—C43A—C44A—C45A	55.8 (3)	C48B—C43B—C44B—C46B	63.4 (2)
C48A—C43A—C44A—C46A	−1.9 (3)	C42B—C43B—C44B—C47B	124.48 (19)
C42A—C43A—C44A—C46A	178.2 (2)	C48B—C43B—C44B—C47B	−56.8 (2)
C48A—C43A—C44A—C47A	113.5 (2)	C42B—C43B—C44B—C45B	5.9 (3)
C42A—C43A—C44A—C47A	−66.5 (2)	C48B—C43B—C44B—C45B	−175.4 (2)
C48A—C43A—C44C—C47C	59.1 (3)	C42B—C43B—C44D—C47D	57.6 (11)
C42A—C43A—C44C—C47C	−120.8 (3)	C48B—C43B—C44D—C47D	−123.6 (11)
C48A—C43A—C44C—C45C	−177.2 (2)	C42B—C43B—C44D—C46D	−176.9 (9)
C42A—C43A—C44C—C45C	2.9 (3)	C48B—C43B—C44D—C46D	1.8 (9)
C48A—C43A—C44C—C46C	−61.8 (2)	C42B—C43B—C44D—C45D	−64.0 (8)
C42A—C43A—C44C—C46C	118.3 (2)	C48B—C43B—C44D—C45D	114.8 (8)
C36A—C35A—C48A—C43A	−0.6 (2)	C36B—C35B—C48B—C43B	0.0 (2)
O3A—C35A—C48A—C43A	172.73 (12)	O3B—C35B—C48B—C43B	173.55 (13)
C42A—C43A—C48A—C35A	1.1 (2)	C42B—C43B—C48B—C35B	−1.6 (2)
C44A—C43A—C48A—C35A	−178.86 (14)	C44B—C43B—C48B—C35B	179.65 (14)
C44C—C43A—C48A—C35A	−178.86 (14)	C44D—C43B—C48B—C35B	179.65 (14)
C4A—O4A—C49A—C50A	−108.44 (14)	C4B—O4B—C49B—C62B	115.94 (15)
C4A—O4A—C49A—C62A	79.36 (16)	C4B—O4B—C49B—C50B	−69.91 (18)
C62A—C49A—C50A—C51A	0.8 (2)	C62B—C49B—C50B—C51B	0.0 (2)
O4A—C49A—C50A—C51A	−170.96 (12)	O4B—C49B—C50B—C51B	−173.66 (13)
C49A—C50A—C51A—C56A	−0.5 (2)	C49B—C50B—C51B—C56B	−0.4 (2)
C49A—C50A—C51A—C52A	175.11 (13)	C49B—C50B—C51B—C52B	177.88 (14)
C56A—C51A—C52A—C54A	−157.37 (16)	C56B—C51B—C52B—C55B	−179.22 (16)
C50A—C51A—C52A—C54A	27.2 (2)	C50B—C51B—C52B—C55B	2.6 (2)
C56A—C51A—C52A—C53A	−36.2 (2)	C56B—C51B—C52B—C54B	−59.1 (2)
C50A—C51A—C52A—C53A	148.38 (16)	C50B—C51B—C52B—C54B	122.70 (18)
C56A—C51A—C52A—C55A	83.50 (19)	C56B—C51B—C52B—C53B	61.0 (2)
C50A—C51A—C52A—C55A	−91.90 (18)	C50B—C51B—C52B—C53B	−117.18 (18)
C50A—C51A—C56A—C57A	0.0 (2)	C50B—C51B—C56B—C57B	0.5 (2)
C52A—C51A—C56A—C57A	−175.55 (16)	C52B—C51B—C56B—C57B	−177.74 (15)
C51A—C56A—C57A—C62A	0.2 (3)	C51B—C56B—C57B—C62B	−0.4 (3)
C51A—C56A—C57A—C58A	179.6 (2)	C51B—C56B—C57B—C58B	177.96 (16)
C51A—C56A—C57A—C58C	179.6 (2)	C51B—C56B—C57B—C58D	177.96 (16)
C62A—C57A—C58A—C60A	−129.7 (6)	C56B—C57B—C58B—C60B	−118.0 (2)
C56A—C57A—C58A—C60A	51.0 (6)	C62B—C57B—C58B—C60B	60.3 (3)
C62A—C57A—C58A—C61A	7.0 (3)	C56B—C57B—C58B—C59B	4.3 (3)
C56A—C57A—C58A—C61A	−172.3 (2)	C62B—C57B—C58B—C59B	−177.5 (2)
C62A—C57A—C58A—C59A	111.9 (3)	C56B—C57B—C58B—C61B	122.9 (2)
C56A—C57A—C58A—C59A	−67.4 (3)	C62B—C57B—C58B—C61B	−58.9 (2)
C62A—C57A—C58C—C59C	143.2 (6)	C56B—C57B—C58D—C60D	−172.4 (10)
C56A—C57A—C58C—C59C	−36.1 (6)	C62B—C57B—C58D—C60D	5.9 (10)
C62A—C57A—C58C—C61C	7.0 (3)	C56B—C57B—C58D—C61D	65.6 (7)
C56A—C57A—C58C—C61C	−172.3 (2)	C62B—C57B—C58D—C61D	−116.2 (7)
C62A—C57A—C58C—C60C	−104.9 (3)	C56B—C57B—C58D—C59D	−48.6 (6)
C56A—C57A—C58C—C60C	75.8 (3)	C62B—C57B—C58D—C59D	129.7 (6)
C50A—C49A—C62A—C57A	−0.5 (2)	C50B—C49B—C62B—C57B	0.1 (2)
O4A—C49A—C62A—C57A	171.16 (14)	O4B—C49B—C62B—C57B	174.11 (13)
C56A—C57A—C62A—C49A	0.0 (2)	C56B—C57B—C62B—C49B	0.0 (2)
C58A—C57A—C62A—C49A	−179.3 (2)	C58B—C57B—C62B—C49B	−178.37 (15)
C58C—C57A—C62A—C49A	−179.3 (2)	C58D—C57B—C62B—C49B	−178.37 (15)

### Hydrogen-bond geometry (Å, º)

**Table:**

*D*—H···*A*	*D*—H	H···*A*	*D*···*A*	*D*—H···*A*
C48*A*—H48*A*···O1*B*	0.95	2.47	3.3187 (17)	149
C50*A*—H50*A*···O4*B*	0.95	2.56	3.4413 (18)	154
C34*B*—H34*B*···O2*A*	0.95	2.55	3.3259 (18)	139
C36*B*—H36*B*···O1*A*	0.95	2.48	3.4101 (18)	165
